# Differential effect of asparagine and glutamine removal on three adenocarcinoma cell lines

**DOI:** 10.1016/j.heliyon.2024.e35789

**Published:** 2024-08-03

**Authors:** Greta Pessino, Leonardo Lonati, Claudia Scotti, Silvia Calandra, Ornella Cazzalini, Ombretta Iaria, Andrea Previtali, Giorgio Baiocco, Paola Perucca, Anna Tricarico, Martina Vetro, Lucia Anna Stivala, Carlo Ganini, Marta Cancelliere, Massimo Zucchetti, Isabella Guardamagna, Maristella Maggi

**Affiliations:** a Unit of Immunology and General Pathology, Department of Molecular Medicine, University of Pavia, 27100 Pavia, Italy; b Laboratory of Radiation Biophysics and Radiobiology, Department of Physics, University of Pavia, 27100 Pavia, Italy; c Division of Medical Oncology, Azienda Ospedaliero Universitaria Consorziale Policlinico di Bari, Bari, Italy; d Interdisciplinary Department of Medicine, A. Moro University of Bari, Bari, Italy; e Rheumatology Unit, Department of Internal Medicine and Medical Therapy, University of Pavia, Pavia, Italy; f Laboratory of Cancer Pharmacology, Istituto di Ricerche Farmacologiche Mario Negri IRCCS, Via Mario Negri 2, Milan, Italy

**Keywords:** Asparaginase, Cell cycle, Adenocarcinoma, Solid tumors, Renal cell carcinoma, Breast carcinoma, Asparagine synthetase, Glutamine synthetase

## Abstract

Asparagine and glutamine depletion operated by the drug Asparaginase (ASNase) has revolutionized therapy in pediatric patients affected by Acute Lymphoblastic Leukemia (ALL), bringing remissions to a remarkable 90 % of cases. However, the knowledge of the proproliferative role of asparagine in adult and solid tumors is still limited. We have here analyzed the effect of ASNase on three adenocarcinoma cell lines (A549, lung adenocarcinoma, MCF-7, breast cancer, and 786-O, kidney cancer). In contrast to MCF-7 cells, 786-O and A549 cells proved to be a relevant target for cell cycle perturbation by asparagine and glutamine shortage. Indeed, when the cell-cycle was analyzed by flow cytometry, A549 showed a canonical response to asparaginase, 786-O cells, instead, showed a reduction of the percentage of cells in the G1 phase and an increase of those in the S-phase. Despite an increased number of PCNA and RPA70 positive nuclear foci, BrdU and EdU incorporation was absent or strongly delayed in treated 786-O cells, thus indicating a readiness of replication forks unmatched by DNA synthesis. In 786-O asparagine synthetase was reduced following treatment and glutamine synthetase was totally absent. Interestingly, DNA synthesis could be recovered by adding Gln to the medium. MCF-7 cells showed no significant changes in the cell cycle phases, in DNA-bound PCNA and in total PCNA, but a significant increase in ASNS and GS mRNA and protein expression. The collected data suggest that the effect observed on 786-O cells following ASNase treatment could rely on mechanisms which differ from those well-known and described for leukemic blasts, consisting of a complete block in the G1/S transition in proliferating cells and on an increase on non-proliferative (G0) blasts.

## Highlights

• Asparaginase alters cell cycle of a renal cell carcinoma cell line.
• Asparaginase alters cell cycle of a lung adenocarcinoma cell line.
• Asparaginase alters DNA synthesis efficiency of a renal cell carcinoma cell line.
• Asparaginase inhibits proliferation of two adenocarcinoma cell lines.
• Asparaginase can have a cytostatic effect on adenocarcinoma cells.

## 1 Introduction

Asparagine (Asn) is not an essential amino acid. Most human healthy cells are able to *de novo* synthetize Asn through Asparagine Synthetase (ASNS), an enzyme which catalyzes the conversion of aspartate and glutamine into asparagine (Asn) and glutamate (Glu) [1]. However, the expression of the *ASNS* gene is strictly regulated and human healthy cells are able to compensate for circulating Asn depletion only through *ASNS* gene upregulation, which is mediated by the integrated stress response (ISR), or the amino acid response (AAR) [2]. AAR is triggered by amino acid deprivation, which results in a general protein synthesis reduction *via* mTOR pathway inhibition [3] and in the promotion of the translation of a subset of mRNAs encoding for a series of key factors. Among them, ATF4 (Activating Transcription Factor 4) is responsible for the upregulation of the *ASNS* gene [3].

Despite being a non-essential amino acid in healthy cells, Asn represents a key metabolite in tumor growth support: it is a robust target in hematological malignancies, a promoter of solid tumor progression [4,5] and a cell signaling regulator in cancer cells [6,7]. Its enzymatic removal has proven to be an essential therapeutic strategy in specific types of cancer. Since its introduction in the 1970s, in fact, the anti-metabolism drug l-Asparaginase has become a key component of the combined chemotherapy scheme used in pediatric Acute Lymphoblastic Leukemia (ALL) treatment, playing a decisive role in increasing complete remissions achieved up to 90% in patients [8].

l-Asparaginase is an amidohydrolase (ASNase, EC 3.5.1.1) with a prevalent asparaginolytic activity, catalyzing the hydrolysis of asparagine (Asn), and a secondary glutaminolytic activity, catalyzing the hydrolysis of glutamine (Gln) [9]. The dependence of leukemia cells on Asn extracellular supply is considered to be the cause of ASNase antitumoral effect [10], with therapeutic asparagine depletion levels achieved at an ASNase activity of ≥0.1 IU/ml in patients’ serum [11]. The reason for this dependency and, in turn, for their sensitivity to ASNase, is mainly identified in their reduced or complete loss of *ASNS* expression, although the precise molecular basis of this phenomenon has not yet been fully elucidated [12]. Besides inhibiting protein synthesis, in fact, ASNase also acts on the regulation of nucleotide synthesis and on cell cycle progression [6]. It is well known that, in leukemic blasts, ASNase affects cell cycle progression, causing a blockade of the G1/S transition in proliferating cells and an increase of non-proliferative (G0) blasts [13].

In contrast, the role of ASNase secondary glutaminolytic activity is strongly debated. It is difficult to determine whether its main contribution is to support ASNase antitumoral effect or to promote the onset of side effects. Indeed, glutamine is the amino acid most organs normally depend on, with a plasma concentration of approximately 600–900 μM (10 times higher than Asn) [14]. Solid evidence has shown that ASNase glutaminolytic activity is essential to sustain ASNase anti-tumoral activity over time [15], given that its depletion prevents the synthesis of Asn (*via ASNS* upregulation) and causes the inhibition of the mTOR pathway [16].

To date, solid tumors are the most difficult to treat and the quest for new therapies is one of the main challenges that cancer research is facing worldwide. In this respect, the metabolic asset of cancer cells is receiving great attention, as tumors typically rewire their metabolism to respond to the need imposed by perpetual growth. Therefore, targeting tumor-specific amino acidic dependency in the Tumor Microenvironment (TME) is becoming an increasingly attractive strategy.

Despite its success in pediatric hematological malignancies, ASNase effectiveness in solid tumors has turned out to be not as remarkable, but worth of further investigation [17].

So far, Phase I and II clinical trials including ASNase-based therapy in the treatment of pancreatic adenocarcinoma have been successfully completed [18] and a new Phase I study is starting recruitment [19]. Promising results have also been obtained in several solid tumor cell lines, such as gastric, breast, lung, and colon cancer [20,21]. Additionally, it has been demonstrated that ASNase plays a crucial role in inhibiting the spread of metastasis in a mouse model of breast cancer [22]. However, in these circumstances, sensitivity to ASNase has proven to be difficult to predict and does not seem to be associated with traditional markers of ASNase sensitivity, such as levels of Asparagine Synthetase (ASNS) and Glutamine Synthetase (GS). Specifically, since adenocarcinomas constitute the majority of cancers in the global population [6], harnessing the therapeutic potential of ASNase in this extensive group of tumors would be extremely valuable [23]. Based on these considerations, in this work we present an explorative study of the response to ASNase treatment of three cell lines representative of epithelial solid tumors, 786-O (primary renal clear cell adenocarcinoma), A549 (alveolar cell carcinoma) and MCF-7 (breast adenocarcinoma). We observed different behaviors in terms of cell-cycle modifications and expression of ASNase sensitivity markers (i.e., ASNS, GS). Particularly, the collected data suggest that, while a canonical response to ASNase can be observed in A549 cells, the inhibitory effect observed in 786-O cells upon ASNase treatment could rely on mechanisms which differ from those well-known and described. Hence, this explorative work lays the foundations for the description of a possible differential mechanism of action of ASNase in solid tumors, which could represent a turning point for further studies aimed at exploring the possibility of using ASNase in the treatment of aggressive solid cancers.

## 2 Materials and methods

### 2.1 Cell culture

786-O cells (kidney, primary clear cell carcinoma, American Tissue Culture Collection, ATCC, Manassas, VA, USA) were cultured in RPMI 1640 medium (Gibco™, ThermoFisher Scientific, Waltham, MA, USA); A549 lung, alveolar epithelial carcinoma, American Tissue Culture Collection, ATCC, Manassas, VA, USA) and MCF-7 cells (breast, metastatic adenocarcinoma, American Tissue Culture Collection, ATCC, Manassas, VA, USA) were cultured in Dulbecco's Modified Eagle's medium (DMEM high glucose, 4.5 g/l glucose, PAN-Biotech GmbH, Aidenbach, Germany). In all cases, medium was supplemented with 10 % fetal bovine serum (FBS, Sigma-Aldrich, St. Louis, MO, USA), 2 mM l-glutamine (EuroClone, Siziano, Italy), 100 U/ml penicillin (EuroClone, Siziano, Italy), 100 μg/ml streptomycin (EuroClone, Siziano, Italy) and cells were cultured at 37 °C in a humidified atmosphere with 5 % CO_2_. Cells were passaged around 85–90 % of confluence, typically every three days.

### 2.2 ASNase production

ASNase needed for treatments was obtained by overexpression and production of the highly stable mutant N24S *E. coli* type II Asparaginase (EcAII) in recombinant form as already described in detail [24]. In general, a dosage ranging from 0.1 to 1 U/ml of Asparaginase proves adequate for ensuring depletion of asparagine and glutamine, both in vitro (i.e., within the culture medium) and *in vivo* (i.e., within patient sera) [[25], [26], [27], [28]], therefore we selected this dose range for our experiments and confirmed its efficacy by amino acid dosage.

### 2.3 Cell cycle analysis

2.5x10^5^ A549, MCF-7 and 786-O cells were seeded in 60 mm Petri dishes. 24 h after seeding, cells were treated with different concentrations of ASNase (0, 0.05, 0.50, 1.0 U/ml). 72 h after treatment, 5-ethynyl-2′-deoxyuridine (EdU, dissolved in DMSO following manufacturer instructions) was added to a final concentration of 10 μM and cells were then incubated at 37 °C in a humidified atmosphere with 5 % CO_2_ for 1 h. After incubation, cells were collected by trypsinization, resuspended by pipetting, fixed with 2 % (w/v) paraformaldehyde for 10 min and permeabilized with 70 % v/v ethanol in 0.9 % w/v NaCl. Fixed and permeabilized cells were incubated with the anti-phospho H3 (Ser 10) primary antibody (1:5000 dilution, Millipore, RRID:AB_310016) at RT for 1 h and then the goat anti-mouse IgG 555 secondary antibody (1:200 dilution, Molecular Probes, RRID:AB_2535846) at RT for 30 min. EdU-positive cells were detected by Click-iT™ EdU Cell Proliferation Kit for Imaging, Alexa Fluor™ 488 dye (Invitrogen™ ThermoFisher Scientific, Waltham, MA, USA). DNA staining was obtained using the FxCycle™ Violet Stain kit (Invitrogen™ ThermoFisher Scientific, Waltham, MA, USA). An Attune NxT Acoustic Focusing flow cytometer (ThermoFisher Scientific, Waltham, MA, USA) was used for the analysis of stained samples. All the collected data were analyzed using the Attune NxT software v February 4, 1627.1. Cell cycle phases analysis was performed using the following gates built on a bi-parametric scatter chart (EdU A488 vs. Fx-cycle A405): G1, diploid (2n) DNA content, negative for EdU; Stot, both EdU positive and negative, DNA content comprised between 2n and 4n; G2, EdU negative, tetraploid (4n) DNA content; M is a subgate of the G2 gate comprising the population positive for the pH3 (S10) marker. Examples of the gating procedure are given in Supplementary Materials (Fig. S1). Data are represented as percentages of total counts of cycling cells. Median Fluorescence Intensity (MFI) at A488 was calculated by subtracting the MFI value of the EdU negative population from the MFI value of the EdU positive population. The obtained values were normalized to the value obtained for the untreated sample. Data were interpolated using the four-parameter logistic curve (Top constraint: 1, Bottom constraint: 0) to obtain IC50 values defined as the EcAII concentration at which MFI is half maximum ± standard error.

### 2.4 Survival assay

The clonogenic survival of 786-O and A549 cells was evaluated plating cells at low density. 24 h after seeding, cells were treated with different concentrations of EcAII (0.05, 0.50, 1.00 U/ml). After 7–8 d from seeding, colonies were fixed and stained with a solution containing 1 % w/v Crystal Violet (Sigma-Aldrich). The day after, colonies were counted. Plate efficiency (PE) was calculated as follow:

$$PE=\frac{number\;of\;colonies\;formed}{number\;of\;cells\;seeded}$$

Data were expressed as Survival Fraction (SF) calculated as follows:

$$SF=\left(\frac{number\;of\;colonies\;formed\;after\;treatment}{number\;of\;cells\;seeded}\right)/PE$$

Data were plotted by non-linear three-parameter equation to obtain the SF50 value defined as the EcAII concentration at which the survival fraction is 0.5.

### 2.5 Proliferation assay

A549, 786-O and MCF-7 cells were seeded at 8x10^4^ cells in 2 ml complete medium in 6-well plates. 24 h after seeding, cells were treated with different concentrations of EcAII (0.0, 0.05, 0.50, 1.00 U/ml). 72 h after treatment, cells were detached from the support and counted with trypan blue. Cell proliferation was evaluated as mean of doubling time using the following formula:

$$Doubling\;per\;day\;\left(d\right)=\frac{\log\left(2\right)\;\left(\frac{count\;at\;the\;end\;of\;treatment}{count\;at\;seeding}\right)}{days}$$

Data were normalized to the untreated control (CTRL = 1). For dose-response evaluation, data were plotted by non-linear three-parameter equation to obtain the IC50 value defined as the EcAII concentration at which the cell number is 0.5.

### 2.6 Continuous Fluorescence Intensity (CFI) assay

2.5x10^5^ 786-O cells were seeded in 60 mm Petri dishes. 24 h after seeding, cells were treated with 1.0 U/ml ASNase. 48 h after seeding, EdU was added to a final concentration of 10 μM. Cells were then incubated at 37 °C in a humidified atmosphere with 5 % CO_2_ for the following timepoints: 1, 3, 5, 6, 7, 8, 9, 10, and 11 h. Fixed and permeabilized cells were stained as previously described. Cell cycle phases analysis was done using the following gates built on a bi-parametric scatter chart (EdU A488 vs. Fx-cycle A405): zero generation G1, EdU negative cells with 2n DNA content; EdU+, EdU positive cells with DNA content between 2n and ≤4n; zero generation G2, EdU negative cells, with 4n DNA content. Data are represented as percentages whit respect to total counts of cycling cells. Peak Fluorescence Intensity (PFI) was calculated by subtracting the peak of fluorescence intensity of the EdU negative population from the one of the EdU positive population. PFI increment was measured by subtracting the initial PFI value (e.g., PFI at 1 h) from the PFI value obtained for each timepoint. The rate of PFI increase was determined by measuring the slope passing through experimental points in the linear phase of the PFI versus time curve (e.g., 1–9 h).

### 2.7 Western blot

4x10^5^ cells were seeded in 60 mm Petri dishes. 24 h after seeding, cells were treated with different concentrations of ASNase (range 0.0–1.0 U/ml). 72 h after treatment, cells were collected by trypsinization, resuspended in 100 μl lysis buffer (Pierce™ RIPA buffer, ThermoFisher) supplemented with cOmplete™, Mini, EDTA-free Protease Inhibitor Cocktail (Roche, Basel, Switzerland), Phosphatase Inhibitor Cocktail 2 (Sigma-Aldrich, Merck KGaA, Darmstadt, Germany). After 20 min incubation on ice with mild shaking, cell debris were removed by centrifugation and total protein content was quantified by a BCA assay (SERVA Electrophoresis GmbH, Heidelberg, Germany). 50 μg total proteins per lane were loaded onto a 12 % acrylamide/bisacrylamide gel and blotted onto a PVDF membrane. The following primary antibodies were used for incubation: PC10 anti-PCNA (1:1000 dilution, Dako, RRID: M0879), anti-actin (1:500 dilution, Invitrogen, RRID: AB_2223196), anti-ASNS (1:1000 dilution, Invitrogen, RRID:AB_2809229), anti-GLUL (1:10000 dilution, Sigma Aldrich, RRID: AB_259853), anti-Vinculin (1:2000 dilution, Sigma Aldrich, RRID:AB_10604160). The following secondary HRP-conjugated antibodies were used: anti-mouse IgG (1:20000 dilution, GE Healthcare, RRID: AB_772210) and anti-rabbit IgG (1:1000 dilution, ThermoFisher Scientific, RRID: AB_228341). Semiquantitative analysis was performed by densitometric analysis using ImageJ v 1.53.

### 2.8 Immunostaining

A549, MCF-7 and 786-O cells were seeded (7x10^4^ cells per well) in 35 mm Petri dishes containing a 22 × 22 mm slide. 24 h after seeding, cells were treated with different concentrations of ASNase (0.50 or 1.00 U/ml). 72 h after treatment, samples were washed with cold PBS and lysed at 4 °C in hypotonic buffer (10 mM Tris-HCl pH 7.4, 2.5 mM MgCl_2_, 0.1 % Nonidet NP-40, 0.2 mM phenylmethylsulfonyl fluoride (PMSF)) for 10 min. Thereafter, samples were washed in PBS, fixed in 4 % formaldehyde for 5 min and permeabilized in 70 % ethanol at −20 °C for 20 min. After blocking, samples were incubated with the primary PC10 anti-PCNA antibody (1:100 dilution, Dako, RRID: M0879) for 1 h and, then, with the secondary anti-mouse Alexa 488 antibody (dilution 1:200, Invitrogen, RRID: AB_141708) for 30 min. Alternatively, cells were incubated with the primary anti-RPA70 antibody (1:100 dilution, Cell Signaling Technologies, RRID:AB_2180506) and then with the secondary anti-rabbit Dylight 594 antibody (1:200 dilution, Abcam, RRID:AB_10680407). DNA was labeled with Hoechst 33258 (1:20.000 dilution). To detect BrdU labeling, cells were incubated with 10 μM BrdU (Merck) before fixation, and then the primary anti-BrdU antibody (1:100 dilution, Cell Signaling Technologies, RRID:AB_10548898) and the secondary anti-mouse Alexa Fluor 488 antibody (1:200 dilution, ThermoFisher Scientific, RRID:AB_2534084) were used. Images were acquired by a confocal TCS SP8 DLS Leica microscope, with 64X magnification and 2.5× zoom. RPA70 nuclei were analyzed with ImageJ v 1.53 analyze particles tool, setting the 0–150 pixel squared range as a limit to define a single focus with a maximal diameter of 0.5 μm. As for PCNA, immunostaining analysis was done by evaluating the ratio of PCNA positive/total nuclei, in order to include all S cells (early, middle and late S cells, which show different staining patterns [29]).

### 2.9 Rescue

A549 and 786-O cells were treated with 1.0 or 0.50 U/ml EcAII, respectively, as previously reported. After 72 h treatment, the medium was added with EdU only (unrescued samples) or EdU and 2 mM L-Gln. In another setting, the medium was replaced with fresh one added with EdU. After 2 h rescue at 37 °C, 5 % CO_2_ cells were fixed and stained as previously reported for cell cycle analysis, except for the M-phase marker anti-phospho H3 which was not considered for this analysis. Data analysis was done by extracting the Median Fluorescence Intensity (MFI) at 488 nm and calculating the difference (EdU positive cells MFI - EdU negative cells MFI). Cell cycle and S phase analysis were done using the gating strategy reported in Supplementary Fig. S4.

### 2.10 Statistics

If not otherwise stated, data were analyzed for their normal distribution by the Shapiro-Wilk test. According to the obtained results, data were analyzed either with one-way ANOVA/*t*-test or with the non parametric Kruskal-Wallis test. The Dunnett's Multiple Comparison test was used to compare means from several experimental groups. Data are means of at least 3 biological replicates (n).

## 3 Results

### 3.1 Asparaginase effect on clonogenic capability, proliferation rate and cell cycle phases

**Fig. 1:**
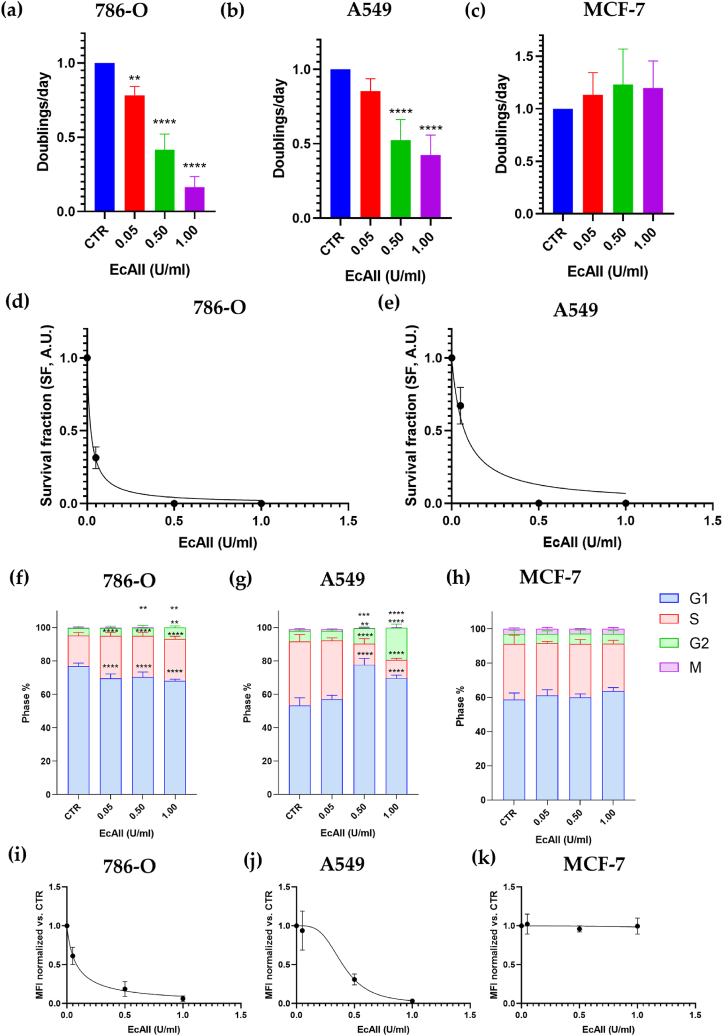
Proliferation and cell cycle analysis of 786-O, A549 and MCF-7 cells treated with 0.00–0.05–0.50 - 1.00 U/ml EcAII for 72 h. **Panels (a)–(c)** proliferation rate of 786-O, A549 and MCF-7 cells. Panels (d) and (e) clonogenic assay of 786-O and A549 cells. **Panels (f)–(h)** 786-O, A549 and MCF-7 cell cycle phases distribution. The Stot gating represents cells with a DNA content between 2n and 4n, both positive and negative for EdU, excluding cells gated in G1 and G2/M. **Panels (i)–(k)** Median Fluorescence Intensity (MFI) of EdU positive cells in 786-O, A549 and MCF-7 cells, respectively. Data were normalized for the untreated CTRL (MFI at 0.00 U/ml = 1). n = 7, *p < 0.05; ** p<0.01; ***p < 0.001.

We evaluated the proliferation efficiency of 786-O, A549, and MCF-7 cells by mean of doublings/day (Fig. 1(a), (b), (c)). The proliferation rate was significantly reduced at the lowest asparaginase dose (0.05 U/ml, proliferation rate 0.758 ± 0,073, p = 0.0043) in 786-O cells with a dose-dependent effect. In A549 cells the proliferation rate was significantly reduced at 0.50 U/ml (0.526 ± 0.136, p < 0,0001). The calculated IC50s were 0.150 ± 0.028 U/ml for 786-O cells and 0.140 ± 0.030 U/ml for A549 cells, respectively (see Supplementary Fig. S2). No significant effect on the proliferation of MCF-7 cells was observed. Given their clear sensitivity to ASNase, 786-O and A549 cells clonogenic capability was evaluated in the absence (CTRL) and in the presence of increasing EcAII doses (Fig. 1(d) and (e)). Already at the lowest EcAII dose (0.05 U/ml), the survival fraction was greatly reduced in 786-O and A549 cells (SF, 0.315 ± 0.074 and 0.671 ± 0.127, respectively) and dropped to 0 at higher doses, with the SF50 being equal to 0.022 ± 0.002 U/ml for 786-O cells and 0.073 ± 0.011 U/ml for A549 cells, respectively.

Analysis of cell cycle phases for 786-O cells (Fig. 1(f), (g), (h)) showed a significant decrease in the G1 population in EcAII treated samples, already at the lowest tested concentration of EcAII (0.05 U/ml); the G2 phase was significantly affected only at the highest tested dose (1 U/ml); the Stot population, which comprise EdU positive and negative cells with a DNA content intermediate between G1 and G2, significantly increased starting from the EcAII lowest dose; the M phase positive population significantly decreased starting from 0.50 U/ml EcAII (Fig. 1(f)). In A549, canonical block of cells in the G1 phase can be observed at 0.50 and 1.0 U/ml EcAII. The cell cycle block in G1 causes the reduction of the percentage of cells in S, M and G2 phase which is significant at 0.50 and 1.0 U/ml (Fig. 1(g)). In the case of MCF-7, no significant changes in cell cycle phases were observed at any EcAII concentration (Fig. 1(h)). Analysis of MFI in the EdU positive population showed a steep decrease in median fluorescence starting from the EcAII lowest dose and with a dose-dependent effect up to 0.50 U/ml in 786-O and A549 cells (Fig. 1(i) and (j)). The calculated synthesis inhibition (SI50) was 0.080 ± 0.009 U/ml for 786-O cells and 0.400 ± 0.066 U/ml in A549 cells. The same analysis done on MCF-7 cells treated with EcAII showed no significant difference even in MFI (Fig. 1(k)).

### 3.2 ASNase effect on the assembly of the replication fork

**Fig. 2:**
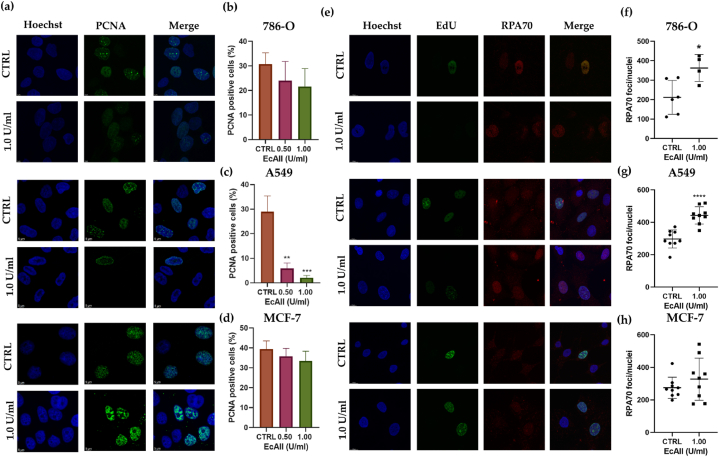
Immunofluorescence (IF) analysis. **Panel (a)** Representative images of control (CTRL) or treated with 1 U/ml EcAII 786-O, A549 and MCF-7 cells stained for nuclear PCNA. Hoechst: blue, PCNA: green. **Panels (b)–(d)** Nuclear PCNA quantification in 786-O, A549 and MCF-7 cells. Data are reported as percentages of PCNA positive cells on total counted cells (n = 3). **Panel (e)** Representative images of 786-O, A549 and MCF-7 cells control (CTRL) or treated with 1 U/ml EcAII and stained for BrdU (green), RPA70 (red) and nuclei (blue). Nuclei counted: 786-O CTRL, 39; 786-O 1U/ml EcAII 14; A549 CTRL, 71; A549 1 U/ml EcAII 48; MCF-7 CTRL, 65; MCF-7 1 U/ml EcAII, 54. **Panels (f)–(h)** Quantification of RPA70 foci in control (CTRL) 786-O cells or treated with 1 U/ml EcAII. n = 3. *: p < 0.05, **: p < 0.01, ***: p < 0.001, ****: p < 0.0001.

Immunostaining of DNA-bound PCNA in cells treated with different doses of EcAII showed a significant reduction in DNA-bound PCNA in A549 cells with a nearly 98 % reduction of PCNA positive cells after treatment with 1 U/ml EcAII (PCNA positive cells, 2.07 % ± 0.97). No significant variations were observed in 786-O and MCF-7 cells even at the highest concentration tested (Fig. 2(a)–(d)).

To further test the efficiency of DNA synthesis in the S-phase, the amount of RPA70, an essential component of DNA replication fork, bound to DNA was analyzed in 786-O, A549 and MCF-7 cells. Following cell lysis, free RPA70 is undetectable, indicating that the observed signal originates from the DNA-bound fraction of RPA70. Additionally, in data analysis we utilized the nuclei as a mask to exclusively capture the fluorescent signal localized in the nucleus. In 786-O cells, from image analysis, it was observed that cells treated with 1 U/ml of EcAII were completely negative for the BrdU signal, but had a higher number of RPA70 positive foci (p = 0.0169) compared to the control cells (Fig. 2(e)-(f)). In A549, in which S-phase is altered by EcAII, a similar increase in RPA70 foci was evident (p < 0.0001) with regard to the untreated control (Fig. 2(e)–(g)). In MCF-7 cells, which cell cycle was not altered by EcAII treatment, no difference in RPA70 foci was evident (p = 0.2967) between control and treated samples(Figure 2(e)-(h)).

### 3.3 ASNase effect on 786-O cell cycle S-phase kinetics

**Fig. 3:**
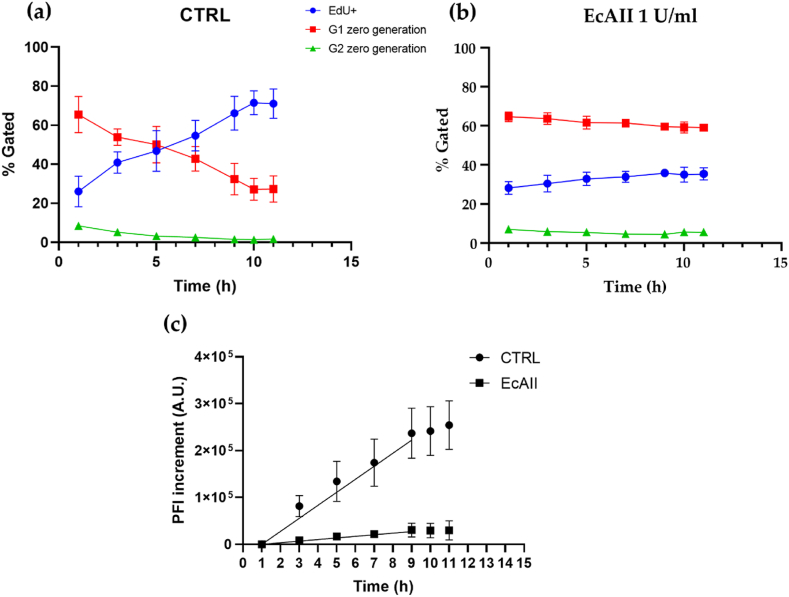
EdU Continuous Fluorescence Intensity (CFI) analysis of 786-O cells. **Panel (a)** and **panel (b)** Percentage distribution of cells into zero generation G1, zero generation G2 and EdU+ in untreated cells and cells treated with 1 U/ml EcAII, respectively. **Panel (c)** Peak Fluorescence Intensity (PFI) increment in untreated cells (CTRL, circle) and cells treated with 1 U/ml EcAII for 48h (square). Lines represent linear regression in the curve linear phase (1–9 h). Data were normalized for the internal zero (PFI at 1 h), (n = 4).

Based on the results obtained by cell cycle analysis, MCF-7 cells were excluded from the analysis of cell-cycle kinetic, because we observed no cell cycle perturbation in the presence of ASNase. Also A549 cells were excluded from this analysis because after treatment with EcAII we could observe an accumulation in G1 without the presence of cells in the EdU negative S-phase population. CFI data clearly showed a slowing down in EdU incorporation in 786-O cells treated with EcAII (1 U/ml, 72 h, Fig. 1(b)). Indeed, the percentage of G1 and G2 cells in the CTRL sample (“zero generation”, i.e., cells in their G1 or G2 phases present at the very beginning of the analysis and not labeled with EdU) gradually decreased upon feeding the culture with EdU, while, in turn, the EdU + population (i.e., cells in S-phase or passed through S-phase and in G1 or G2 phases), increased over time (Fig. 3(a)). A similar behavior was not evident in EcAII treated 786-O cells: no significant changes in the relative percentages of cells in the different phases are evident over time (Fig. 3(b)). These data were confirmed by the rate of Peak Fluorescence Intensity (PFI) increase in the first 9 h of the analysis, which was equal to 27751 ± 1080 A U./h for the CTRL and to 3438 ± 343.4 A U./h in the treated group (Fig. 3(c)).

### 3.4 Western blot analysis

**Fig. 4:**
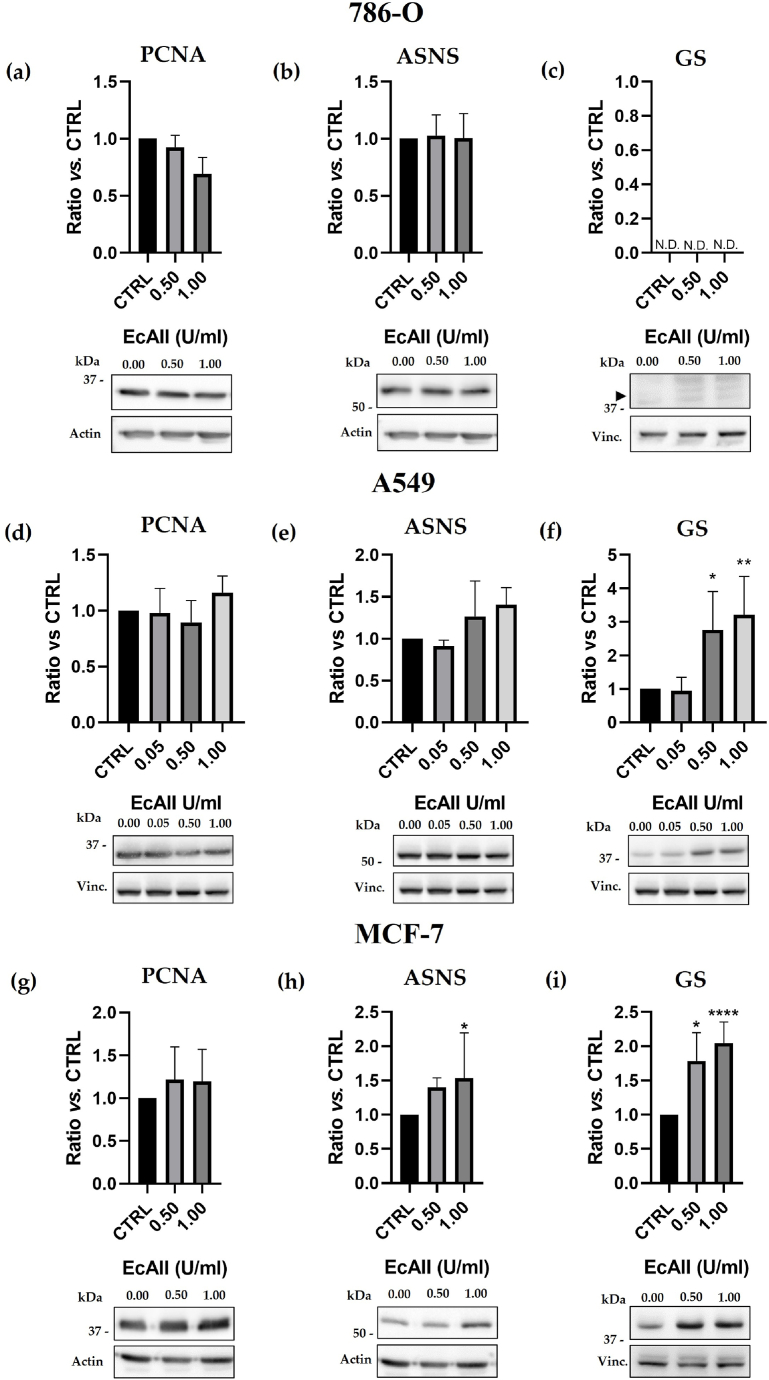
Western blot analysis of PCNA (**panels (a), (d), (g)**), ASNS (**panels (b), (e), (h)**) and GS (**panels (c), (f), (i)**). Representative Western blot membranes for Asparagine synthetase (ASNS), Glutamine synthetase (GS), PCNA, actin or vinculin (Vinc.) are reported in each panel. Densitometric analysis for each target protein is reported in each panel. Data are normalized for the untreated control (CTRL). N.D. indicates “Not Detectable”. ► indicates the expected molecular weight for GS. n = 4, *: p < 0.05; **: p < 0.01, ****: p < 0.0001. Full, non-adjusted images are provided as supplementary material.

No significant dose-dependent change in tPCNA expression was observed in 786-O, A549 and MCF-7 cells (Fig. 4(a)–(d), (g)). As reported in Fig. 4(b) and (e), 786-O and A549 cells showed no significant reduction of ASNS expression at the highest dose of treatment (1 U/ml). In MCF-7 (Fig. 4(h)), a significant increase of ASNS expression can be detected at the highest dose of treatment (1 U/ml, p = 0.04), along with a significant increment of GS expression at 1 U/ml (p = 0.03, Fig. 4(i)). In 786-O a low-to-absent basal or dose-dependent expression of GS was observed (Fig. 4(c)). In A549, instead, a low basal expression of GS in the CTRL was compensated by a significant increase in GS expression in treated samples starting from 0.50 U/ml EcAII (p = 0.03, Fig. 4(f)). GS expression in CTRL samples was evaluated also at the mRNA level by RT-PCR, which confirmed low-to-absent mRNA signal in 786-O and A549, significantly different from the higher mRNA levels detected in MCF-7 cells (p < 0.001, Supplementary Tables S1 and S2 and Supplementary Fig. S3).

### 3.5 Rescue after ASNase treatment in 786-O cells

**Fig. 5:**
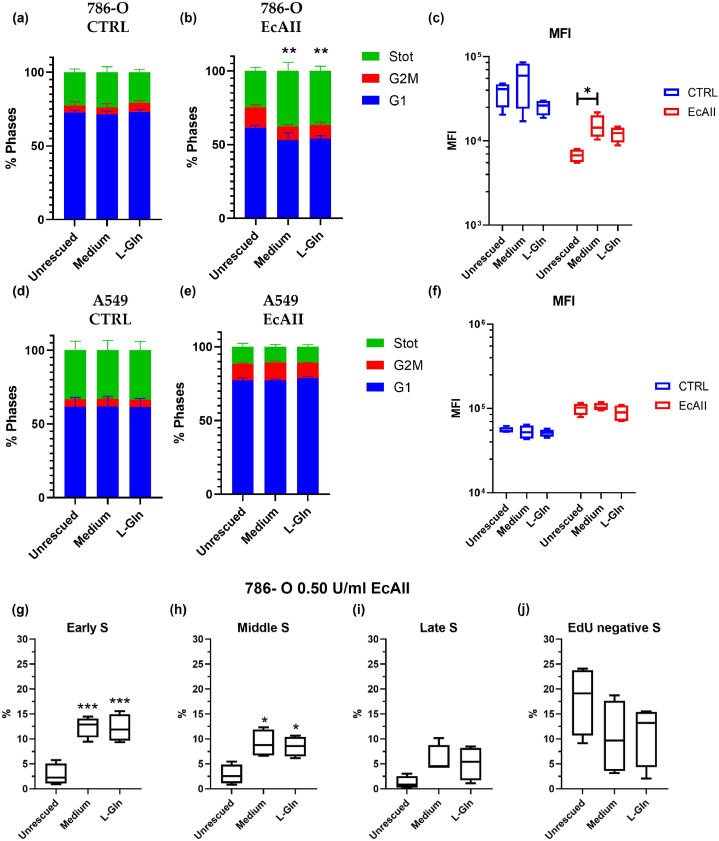
786-O and A549 cells rescue with complete medium (Medium) or 2 mM L-Gln (L-Gln) after EcAII treatment. Panels (a) and (b) and (d) and (e): Cell cycle analysis of control samples and samples treated with 0.50 U/ml EcAII (786-O) or 1.00 U/ml (A549), respectively. Panels (c) and (f) MFI analysis of control (blue) and EcAII-treated (red) samples.786-O and A549 cells, respectively. Panels (g) to (j) S phase analysis in EcAII-treated samples. n = 4, *: p < 0.05; **: p < 0.01, ***: p < 0.001.

To evaluate the recovery of DNA synthesis in 786-O and A549 cells after 72 h of EcAII, the cell cycle of the samples was analyzed after 2 h incubation in EcAII conditioned medium supplemented with 2 mM L-Gln or after replacement of the supernatant with complete medium (Fig. 5). To confirm the complete removal of Asn and Gln in the medium of treated samples, Asn and Gln concentration was evaluated by HPLC method (Supplementary Table 3). In 786-O and A549 cells, analysis of cell cycle phases and Median Fluorescence Intensity (MFI) showed no changes in the control samples in any of the tested conditions (Fig. 5(a) and (d)). A similar effect was observed in A549 cells treated with 1 U/ml EcAII; indeed, the cell cycle phases were not changing in rescued samples if compared to the unrescued one (Fig. 5(e)). Similarly, the MFI values were not changing (Fig. 5(f)). Instead, in 786-O cells treated with EcAII, there was a significant increase in the percentage of cells in the S phase in samples rescued with complete medium or L-Gln (Fig. 5(b)). MFI analysis revealed a significant increase in fluorescence in 786-O cells rescued with complete medium without EcAII, while with the addition of L-Gln in the presence of EcAII, MFI only tends to increase (p = 0.10, Fig. 5(c)). In the control samples, there was no obvious difference between rescued and unrescued samples (Fig. 5(c)-(f)). To further analyze the changes in the S phase population, the percentage of cells in early, middle, late (see Supplementary Fig. S4 for gating) and EdU negative S phase was analyzed. In control samples, no difference was observed between rescued and unrescued cells (Fig. 5(g-j)). In samples treated with EcAII, there was a significant increase in percentage of cells in early and middle S phase (Fig. 5(g) and (h), respectively) for samples rescued with complete medium or with L-Gln. No significant changes were evident in the percentage of cells in late and EdU negative S phase (Fig. 5(i) and (j)).

## 4 Discussion

Cancer treatment is still challenging, mostly in the case of cancers not or only partially responding to classical therapeutic schemes. Combination therapy using different drugs is, presently, the most effective strategy to overcome single-drug treatment resistance [30]. One of the greatest vulnerabilities of tumoral cells is their high and constant request for nutrients support, which results in an increased metabolic demand needed to support cell growth and the increase in cellular mass [[31], [32], [33], [34], [35]]. ASNase is a highly successful, one-of-its-kind bacteria-derived protein drug which works by taking advantage of cancer cells dependency on extracellular Asn supply [35,36]. Canonically, ASNase efficacy is linked to the mTOR mediated block of protein synthesis, dependent on the absence of Asn-loaded tRNA which, in turn, depends on the inability of leukemia cells to produce *ex novo* Asn because of their phenotype deficient in Asparagine Synthetase (ASNS), the only enzyme capable of synthesizing Asn [37,38]. Presently, such a one-way mechanism of action has been put into discussion, mostly because of the growing evidence of a wider role of Asn in several cellular activities [6,12].

ASNase efficacy in solid tumors has been poorly characterized so far, because of its reduced efficacy as a single-agent drug. We and others have recently reported in vitro and/or in vivo evidence of ASNase efficacy on cancer cells derived from solid tumors when combined with other anticancer treatments [39,40]. Starting from these observations, we have tested the efficacy of ASNase in three adenocarcinoma cell lines, A549, MCF-7 and 786-O. MCF-7 cells were selected because of their high similarity with the normal mammary epithelium, reduced invasiveness and high sensitivity to drugs, ideal features to study drug sensitivity or resistance [41,42]. The 786-O cell line was established from a clear cell renal carcinoma [43], which is the most common subtype of RCC, representing approximately 70–80 % of cases. This makes it a representative model for studying the pathogenesis and biology of this prevalent form of kidney cancer [43,44]. The 786-O cell line harbors mutations commonly found in RCC, including loss of the von Hippel-Lindau (VHL) tumor suppressor gene. Mutations in VHL are a hallmark feature of clear cell RCC and play a crucial role in its pathogenesis, making the 786-O cell line a relevant model for studying VHL-deficient RCC [45]. Moreover, renal cell carcinoma (RCC) is viewed as a metabolic disorder stemming from mutations in genes crucial for cellular metabolism [46]. Due to the significance of metabolic changes in RCC, we identified it as a promising candidate for assessing the impact of the antimetabolite drug asparaginase. The A549 cell line is a prominent model in lung cancer research, the cells mimic well the respiratory epithelium and are regarded for their well-characterized biological behavior, mostly at the metabolic level [47]. We discovered that 786-O and A549 cells were highly sensitive to ASNase-mediated growth inhibition, while MCF-7 resulted to be resistant to ASNase inhibition even at high doses.

The effect of Asn deprivation on the cell cycle has always been described as a blockade of the G1/S transition, with cell accumulation in the G0 phase and progressive depletion of cells from the S and, eventually, the M phase [13,48]. Accordingly, we analyzed the cell cycle phases distribution in A549, 786-O and MCF-7 cells. In A549 we observed a canonical accumulation of cells in the G1-phase after treatment with EcAII, which corresponds to a reduction of cells in the S- and G2/M-phases. Surprisingly, in 786-O we observed a reduction in G1 cells corresponding to an increase in cells in the S phase, that were, however, negative for neo-synthesized DNA upon EdU addition. New DNA synthesis inhibition was evident already at low ASNase doses, with a calculated 50 % synthesis inhibition (SI50) equal to 0.08 ± 0.009 U/ml. ASNase treatment greatly affected also A549 and 786-O clonogenic efficiency and proliferation rate, a medium-long term behavior compatible with the slowing down of the cell cycle observed in the initial 72 h.

To have a preliminary knowledge on the mechanism underlying 786-O peculiar response to ASNase treatment, we evaluated the capability of ASNase-treated 786-O cells to properly assemble the replicative fork by immunostaining of PCNA and RPA70, two main proteins involved in the fork assembly and maintenance [49,50]. Both PCNA and RPA70 were found to be properly located on DNA. Moreover, the PCNA nuclear distribution pattern in 786-O cells treated with 1 U/ml ASNase was inhomogeneous, with the prevalence of the middle S phase, as opposed to the CTRL cells, where the S phase distribution was more homogenous as expected in proliferating cells. In A549, as expected the reduction of cells in S-phase corresponded to a significative reduction in the percentage of PCNA positive cells. RPA70 is part of the heterotrimeric replication protein A complex. During DNA replication or repair, it binds and stabilizes single stranded DNA. Interestingly, RPA70 was abundantly present in both A549 and 786-O cells treated with ASNase, even in the absence of newly synthesized DNA (detected as BrdU signal). In 786-O, we can speculate that the presence of PCNA and RPA70 bound to DNA indicate that the replication fork was properly assembled, but elongation of the new DNA strand was impaired; indeed, it is known that if replication forks are stalled, DNA synthesis slows down up to a complete stop [51]. In this case, very likely the DNA polymerase is unable to incorporate dNTPs, while the helicase keeps unwinding the DNA to produce an excess of single stranded DNA and a consequent increase in RPA-coated DNA. In 786-O, the absence of proper pro-apoptotic signals after the stalling of the replicative fork is very likely linked to the homozygous mutation of the *TP53* gene, resulting in the expression of an inactive p53 protein [52].

Given its role in the cell S phase regulation, we have also analyzed total PCNA content in Western blot. Total PCNA is present in reduced amounts in non-proliferating cells and is synthesized in high amounts during the S phase [50]. In 786-O cells, we observed no significant change in PCNA expression, with a non-significant trend to reduction at high dose (1 U/ml), indicating that, very likely, the observed S phase perturbation is not linked to PCNA regulation and availability.

Accordingly, we hypothesized a slowing down in the S phase progression in 786-O cells directly dependent on ASNase treatment. To test our hypothesis, we performed a continuous EdU labeling experiment comparing CTRL and ASNase treated cells to determine EdU incorporation rate. The obtained results showed that DNA synthesis is not completely blocked in treated samples, but in the first 9 h of the analysis the reaction rate is 10-times lower than in the control group, which in turn, slows down the whole cell cycle progression.

To further dissect the molecular features of 786-O cells that make them sensitive to ASNase, we evaluated the expression of the canonical marker of ASNase resistance, ASNS [37]. 786-O, as well as A549 and MCF-7, cells do express basal ASNS, but 786-O and A549 cells, differently from MCF-7, do not respond to ASNase treatment by increasing ASNS expression. In cells resistant to ASNase, ASNS is frequently found to be overexpressed in response to Asn deprivation, and A549 and 786-O incapability to overexpress ASNS strengthens the evidence of their sensitivity to ASNase. MCF-7 dose-dependent overexpression of ASNS matches, instead, with features of ASNase resistance observed in the previous experiments.

Another interesting trait of 786-O cells is their low-to-absent expression of glutamine synthetase (GS), the enzyme involved in glutamine biosynthesis. Indeed, Gln is the secondary target of ASNase even though with a greatly reduced catalytic efficiency. Nevertheless, GS over-expression has been described as one of the primary mechanisms of ASNase resistance together with ASNS overexpression [53]. Indeed, a similar behavior can be observed in MCF-7 ASNase-resistant cells that show a dose-dependent significant increment of both ASNS and GS expression. In A549 cells, instead, we can observe a dose-dependent increase in GS expression upon treatment, which, together with the absent over-expression of ASNS, points to a main role of Asn deprivation in this cell line sensitivity to ASNase and, more in general, in inhibiting cell proliferation as proved also by other studies on the role of ASNS in A549 proliferation [[54], [55], [56], [57]]. This behavior fits well with the observed canonical response to ASNase treatment.

Gln and Asn share the unique characteristic of presenting an amide group in their respective side chains, which makes these non-essential amino acids the primary source of amide groups in bioreactions. Indeed, Gln addiction has been described in several cell lines, and, besides its role as a precursor for trichloroacetic acid cycle (TCA) intermediates and in protein synthesis, Gln role in limiting reactions needed for the biosynthesis of nucleotides is becoming more and more evident. Indeed, purine biosynthesis comprise the reaction catalyzed by amidophosphoribosyltransferase (ATase, 2.1.2.14) which uses Gln as an amide group donor to produce 5-phospho-beta-d-ribosylamine, with a K_m_ for L-Gln in the millimolar range (1.0–1.5 mM) [58]. Shortage of Gln and, alternatively, of Asn [5], caused by ASNase makes this reaction rate-limiting in purine biosynthesis and can cause adenine and guanine shortage that, in turn, can cause DNA replication stalling as well as blocking mRNA synthesis. A similar effect was observed in human keratinocytes cells conditionally auxotrophic for serine (Ser) [59], in which the absence of extracellular Ser supply blocked cell proliferation by limiting purines bioavailability.

To verify the capacity of Gln to correct the observed inhibition of DNA synthesis in 786-O cells, we performed L-Gln rescue experiments. At this stage, we excluded Asn rescue because of ASNase higher catalytic efficiency towards its primary substrate which would make anyway Asn unavailable for the cells [24]. Indeed, supplement of Gln after ASNase treatment can significantly recover DNA synthesis in the short term (2 h). In particular, an increase in the percentage of cells in early and middle S phase can be observed, even in the presence of ASNase. These data indicate that Gln can rescue 786-O cells undergoing *ex novo* DNA synthesis, but not cells blocked in the late S-phase. Indeed, the percentage of cells in the EdU negative S-phase does not change after rescue, and a similar behavior is observed in cells rescued with complete medium.

This observation indicates a further level of complexity in the mechanism of cell-cycle inhibition induced by ASNase, which suggests the need to carefully study the best combination of drugs to be adopted for chemotherapy.

## 5 Conclusions

The present study shows differential effects of asparagine and glutamine removal on three adenocarcinoma cell lines, two sensitive and one insensitive to ASNase action. The relevance of the presented data relies on the description for the first time of a novel effect of amino acids starvation in 786-O cells. Our data confirm the importance of analyzing solid tumor cell lines in vitro for metabolic addictions and the potential of enzyme-based therapy. Further studies will be required to better understand which specific pathway is limited by a low-to-absent Asn and Gln availability and how this different mechanism can hopefully favor the design of more effective combinatorial therapies for the treatment of solid tumors.

## Funding

This research was funded by: “Associazione Gian Franco Lupo—Un sorriso alla vita—ONLUS”; a grant from the 10.13039/501100003407Italian Ministry of Education, University and Research (10.13039/501100003407MIUR) to the 10.13039/501100020258Department of Molecular Medicine, University of Pavia, under the initiative “Dipartimento di Eccellenza (2018–2022)”; funds of the Physics Department, 10.13039/501100004769University of Pavia. This publication is distributed under the terms of open access policies implemented by the Italian Ministry of Education, University and Research (MIUR).

## CRediT authorship contribution statement

**Greta Pessino:** Methodology, Investigation, Formal analysis, Data curation, Conceptualization. **Leonardo Lonati:** Writing – review & editing, Methodology, Investigation, Formal analysis, Data curation, Conceptualization. **Claudia Scotti:** Writing – review & editing, Supervision, Resources, Formal analysis. **Silvia Calandra:** Investigation. **Ornella Cazzalini:** Writing – review & editing, Methodology, Formal analysis. **Ombretta Iaria:** Writing – review & editing, Methodology, Investigation, Data curation. **Andrea Previtali:** Methodology, Investigation. **Giorgio Baiocco:** Writing – review & editing, Supervision, Resources, Formal analysis. **Paola Perucca:** Writing – review & editing, Methodology, Investigation, Formal analysis, Data curation. **Anna Tricarico:** Writing – review & editing, Methodology, Investigation. **Martina Vetro:** Methodology, Investigation. **Lucia Anna Stivala:** Writing – review & editing, Supervision, Investigation, Formal analysis, Data curation, Conceptualization. **Carlo Ganini:** Writing – review & editing, Conceptualization. **Marta Cancelliere:** Investigation. **Massimo Zucchetti:** Methodology, Investigation. **Isabella Guardamagna:** Writing – review & editing, Methodology, Investigation, Formal analysis, Data curation, Conceptualization. **Maristella Maggi:** Methodology, Investigation, Formal analysis, Data curation, Conceptualization.

## Declaration of competing interest

The authors declare the following financial interests/personal relationships which may be considered as potential competing interests:Maristella Maggi has patent A HIGHLY STABLE, PROTEASE-RESISTANT E. COLI ASPARAGINASE issued to University of Pavia. Claudia Scotti has patent A HIGHLY STABLE, PROTEASE-RESISTANT E. COLI ASPARAGINASE issued to University of Pavia. If there are other authors, they declare that they have no known competing financial interests or personal relationships that could have appeared to influence the work reported in this paper.
