# Supplementary material for: Differential effect of asparagine and glutamine removal on three adenocarcinoma cell lines
Source: Heliyon. 2024 Aug 3;10(15):e35789. doi: 10.1016/j.heliyon.2024.e35789 (PMC11337022; doi:10.1016/j.heliyon.2024.e35789)
Supplement: Multimedia component 1 [file mmc1.docx]

**Materials and Methods**

**RT-PCR**

1x10^6^ cells were seeded in 90 mm Petri dishes. 24 h after seeding, cells were treated with 1 U/ml Asparaginase. 72 h after treatment, cells were collected by trypsinization and used for total RNA extraction, which was performed using the GenElute™ Mammalian Total RNA Miniprep Kit (Sigma Aldrich, St. Louis, MO, USA) extraction kit following the manufacturer instructions. Extracted RNA was quantified by spectrophotometric reads (PolarStar Omega, BMG Labtech) and purity was evaluated by means of 260/280 and 260/230 ratios. One μg RNA was used for reverse transcription using the High Capacity cDNA Reverse Transcription Kit (Applied Biosystems, Invitrogen™, ThermoFisher Scientific, Waltham, MA, USA). The obtained cDNA was used as a template to set up real time PCR reactions using Power SYBR Green ® PCR Master Mix (Applied Biosystems, Invitrogen™, ThermoFisher Scientific, Waltham, MA, USA) and CFX BioRad thermocycler (Bio-Rad Laboratories, Hercules, California, USA). Primers used for amplification are listed below.

| Supplementary Table 1. List of RT-PCR primers. | | |
| --- | --- | --- |
| **Gene** | **Forward** | **Reverse** |
| Human GLUL | CTGCCATACCAACTTCAGCACC | ATAGGCACGGATGTGGTACTGG |
| Human GAPDH | GTCTCCTCTGACTTCAACAGCG | ACCACCCTGTTGCTGTAGCCAA |

**Asparagine and glutamine measurement in medium**

The analytical procedure for amino acids quantification in supernatant and medium was carried as previously reported by Gentili D. et al. (“Determination of L-asparagine in biological samples in the presence of L-asparaginase”, J Chromatogr B Biomed Appl. 1994;657(1):47-52. doi:10.1016/0378-4347(94)80068-5). Briefly, 0.06 ml of samples were added with omocysteic acid as internal standard and derivatized with ortho-Phthalaldehyde and quantified by HPLC coupled to fluorescence detector.

**Statistics**

Real-time data were analyzed for their normal distribution by the Shapiro-Wilk test. According to the obtained results, data were analyzed using one-way ANOVA to determine statistical significance (n=4).

**Results**

**RT-PCR**

Glutamine synthetase (GS) mRNA expression levels (Supplementary Table 1 and Supplementary Figure 2) in 786-O and A549 cells resulted to be significatively lower than in MCF-7 cells (n=4, p<0.001).

| Supplementary Table 2. GS mRNA expression levels. | |
| --- | --- |
| **Cell line** | **mRNA expression levels (a.u.)** |
| 786-O | 0.76±0.12 |
| A549 | 0.90±0.38 |
| MCF-7 | 19.41±5.42 |

**Asparagine and glutamine measurement in medium**

| Supplementary Table 3. Asn and Gln levels measurement in medium. | | |
| --- | --- | --- |
| **Sample** | **Asparagine** | **Glutamine** |
| **786-O CTR** | 152.23±6.80 µM | 8.80±3.40 µM |
| **786-O EcAII** | BLOQ | 1.50±2.60 µM |
| **A549 CTR** | BLOQ | 54.30±12.80 µM |
| **A549 EcAII** | BLOQ | BLOQ |
| **MCF7 CTR** | 8.10±1.10 µM | 793.00±178.70 µM |
| **MCF7 EcAII** | BLOQ | 10.00±1.50 µM |
| BLOQ: below limit of quantification which is 0.1 µM for Asn and 5 µM for Gln. | | |


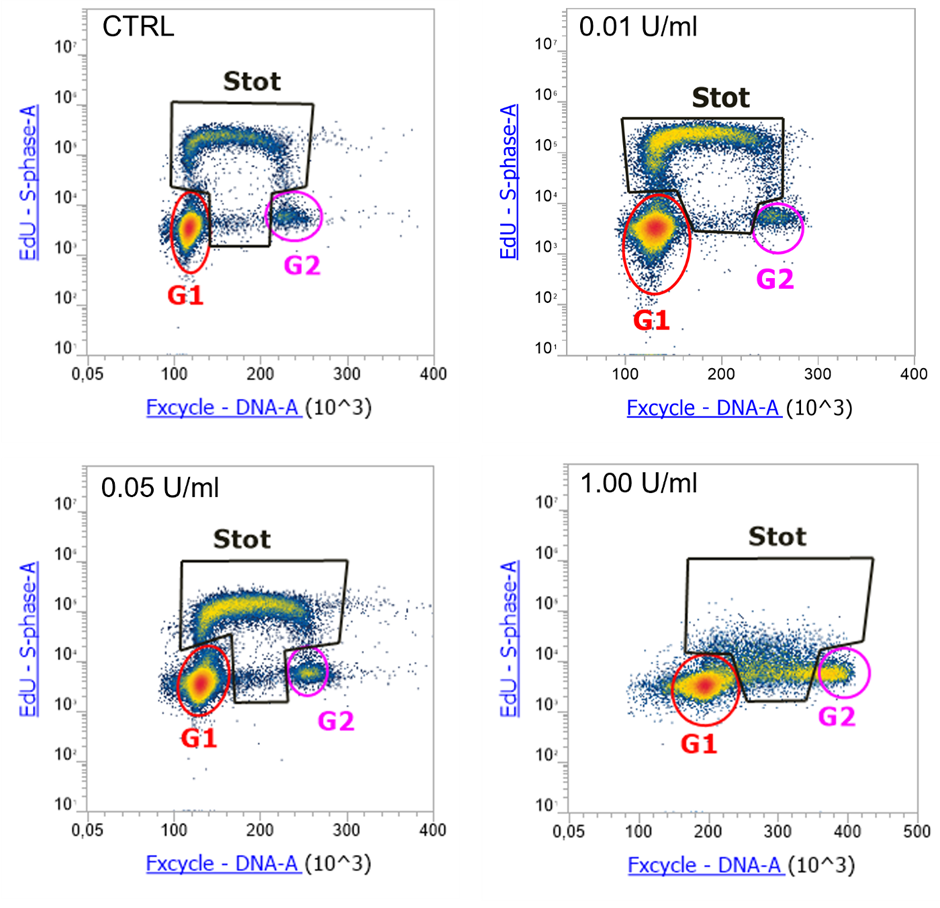


Figure S1. Cell cycle analysis gating. Cell cycle phases analysis was performed using the following gates built on a bi-parametric scatter chart (EdU A488 vs. Fx-cycle A405): G1, diploid (2n) DNA content, negative for EdU; Stot, both EdU positive and negative, DNA content comprised between 2n and 4n, ; G2, EdU negative, tetraploid (4n) DNA content.


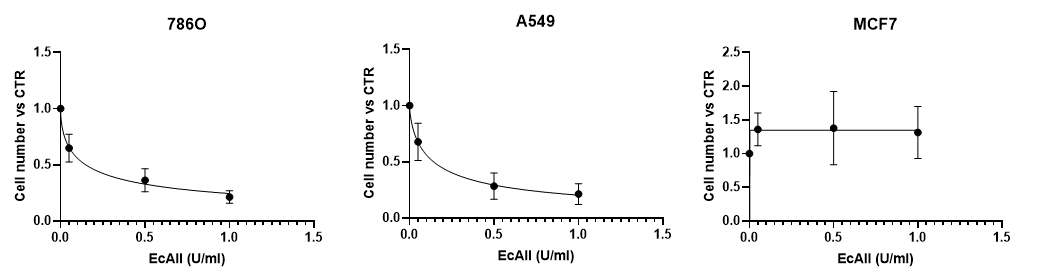


Figure S2. 786-O, A549 and MCF-7 cells dose-response curves used for IC50 calculations.

Figure S3 Glutamine synthetase mRNA expression levels in 786-O, A549 and MCF-7 cells. n=4. ***p<0.001

**
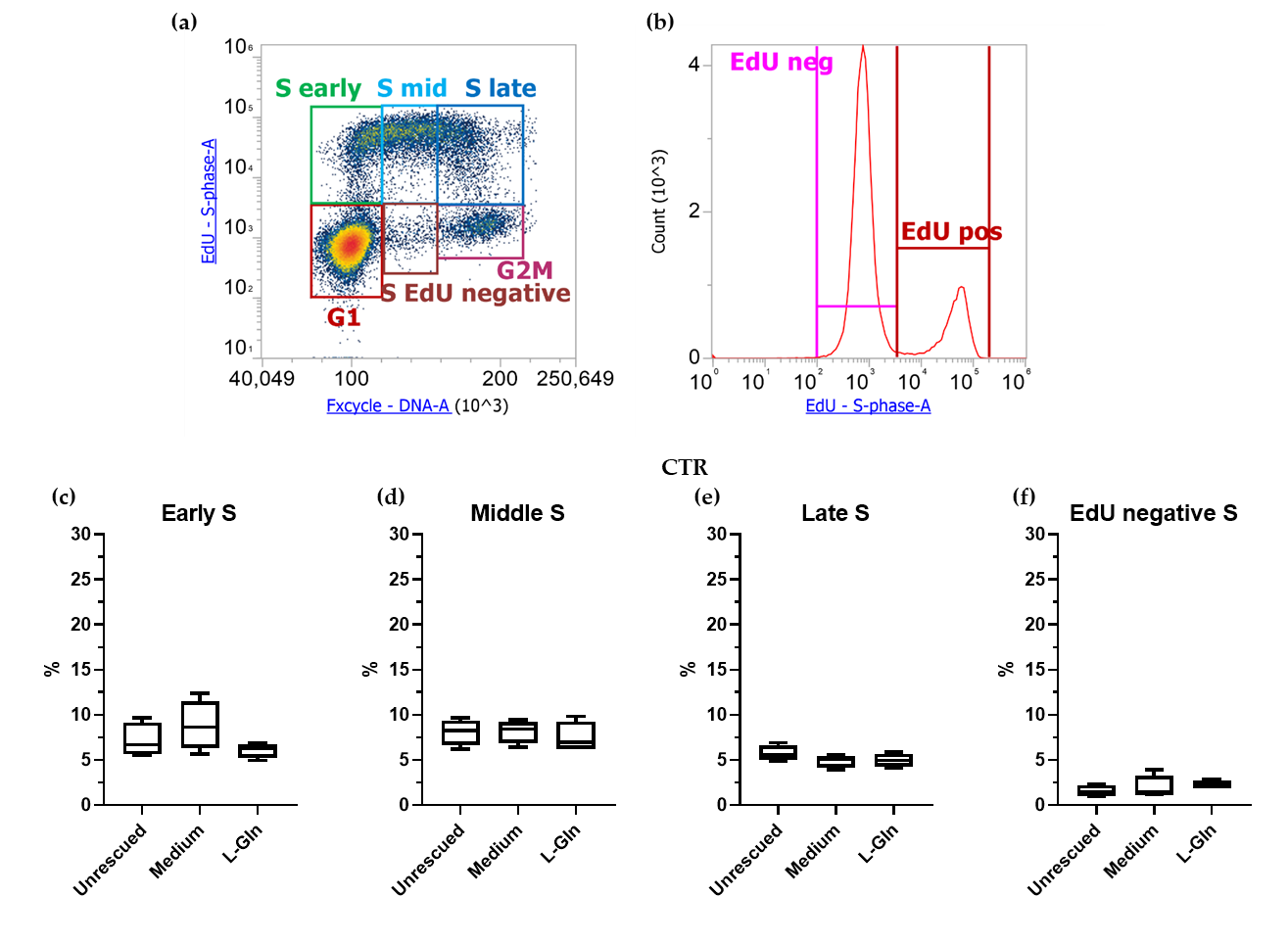
**

Figure S4 Panels (a) and (b) Cell cycle analysis after rescue gating. Panels (c) to (f) S phase analysis in CTR samples.

**Bibliography**

Gentili D, Zucchetti M, Conter V, Masera G, D'Incalci M. Determination of L-asparagine in biological samples in the presence of L-asparaginase. J Chromatogr B Biomed Appl. 1994 Jul 1;657(1):47-52. doi: 10.1016/0378-4347(94)80068-5. PMID: 7952083.

**ORIGINAL WESTERN BLOT**

**
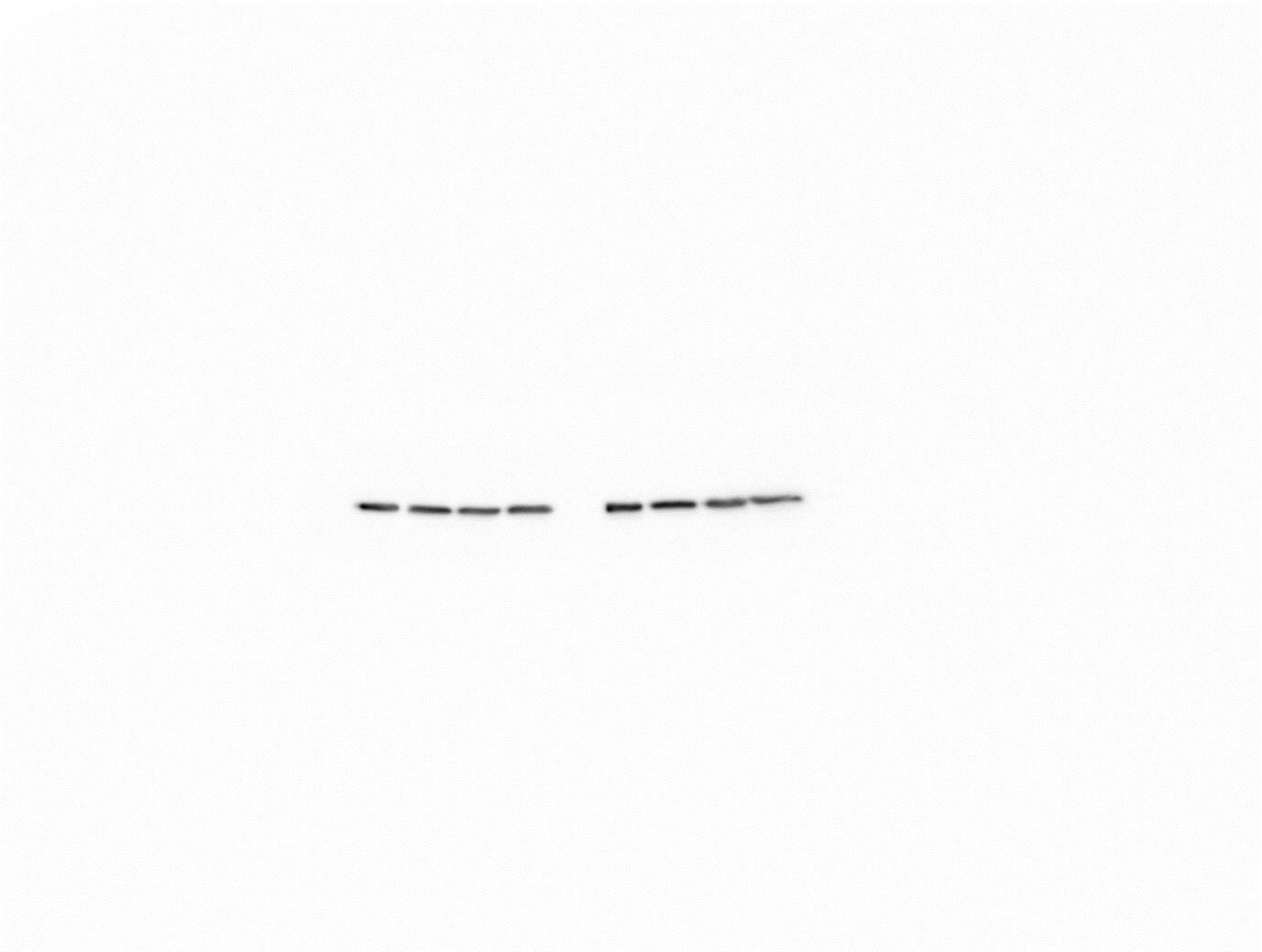
**

**Figure 4 (a) in the manuscript.**

**PCNA, 786-O cell lysate.** Lane 1: MW marker; Lane 2: CTRL; lane 3: 0.5 U/ml; lane 4: 1 U/ml; lane 5: 3 U/ml, lane 6: MW marker; lane 7: CTRL; lane 8: 0.5 U/ml; lane 9: 1 U/ml, lane 10: 3 U/ml.


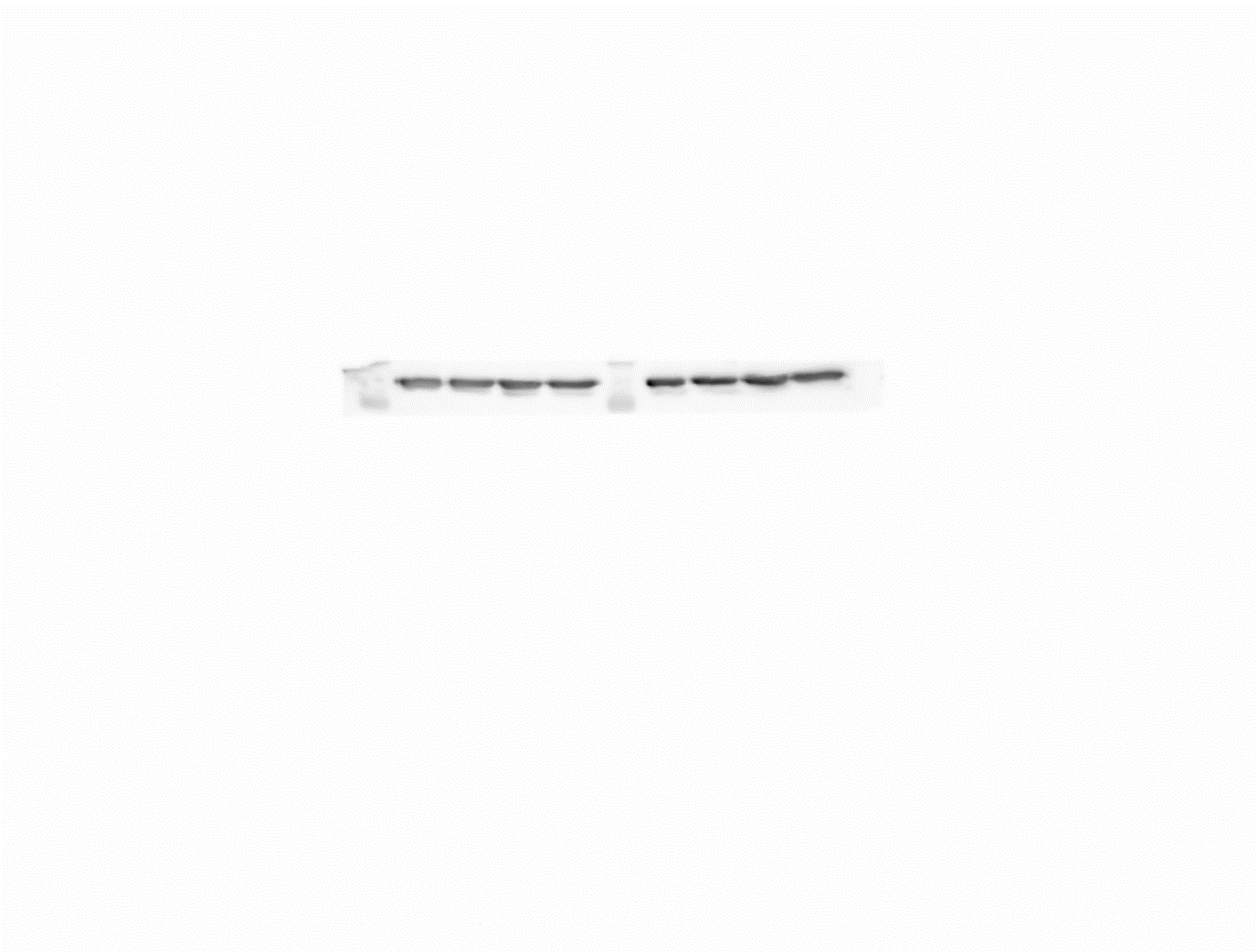


**Figure 4 (a) in the manuscript.**

**ACTIN, 786-O cell lysate.** Lane 1: MW marker; Lane 2: CTRL; lane 3: 0.5 U/ml; lane 4: 1 U/ml; lane 5: 3 U/ml, lane 6: MW marker; lane 7: CTRL; lane 8: 0.5 U/ml; lane 9: 1 U/ml, lane 10: 3 U/ml.

**
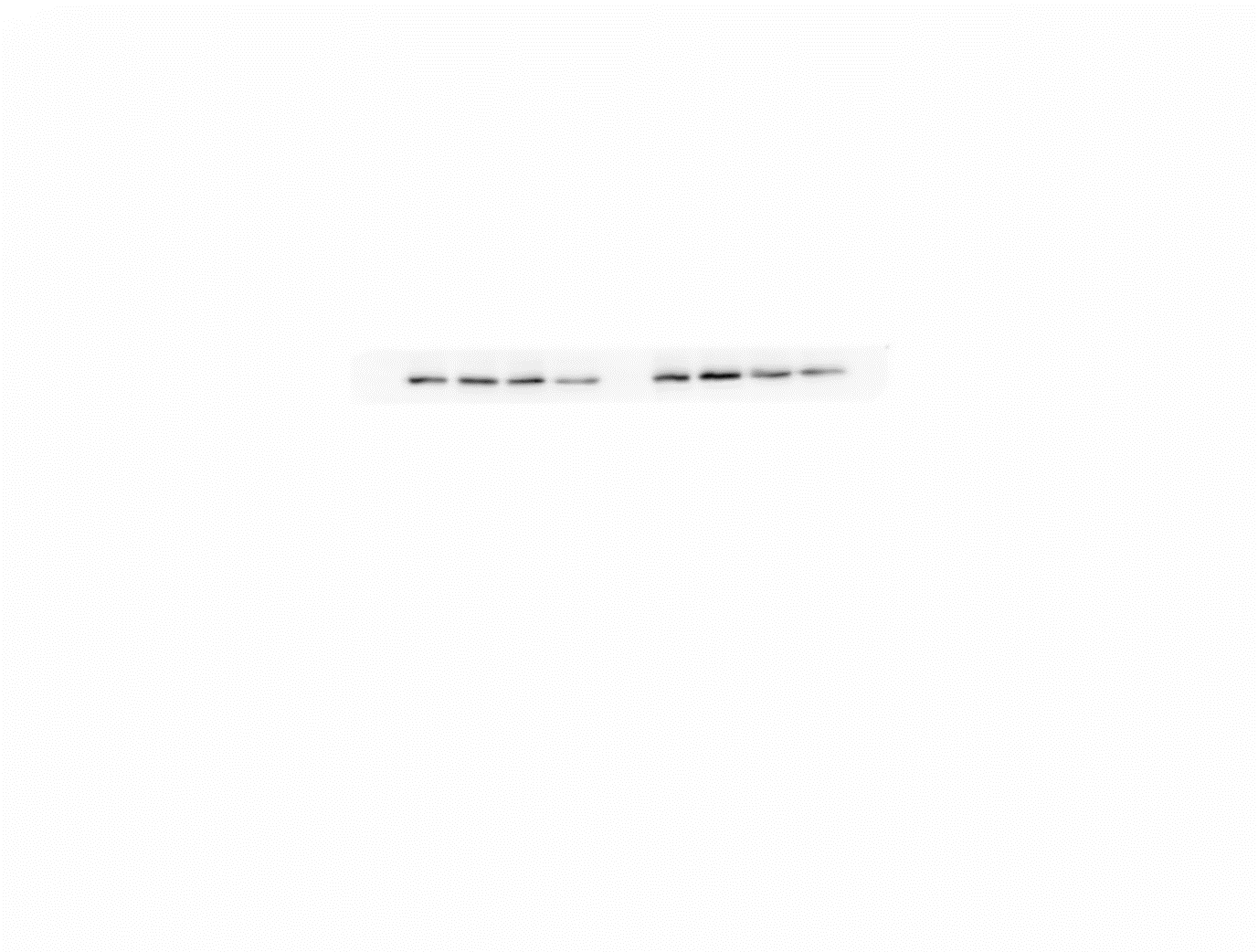
**

**Figure 4 (b) in the manuscript.**

**ASNS, 786-O cell lysate.** Lane 1: MW marker; Lane 2: CTRL; lane 3: 0.5 U/ml; lane 4: 1 U/ml; lane 5: 3 U/ml, lane 6: MW marker; lane 7: CTRL; lane 8: 0.5 U/ml; lane 9: 1 U/ml, lane 10: 3 U/ml.


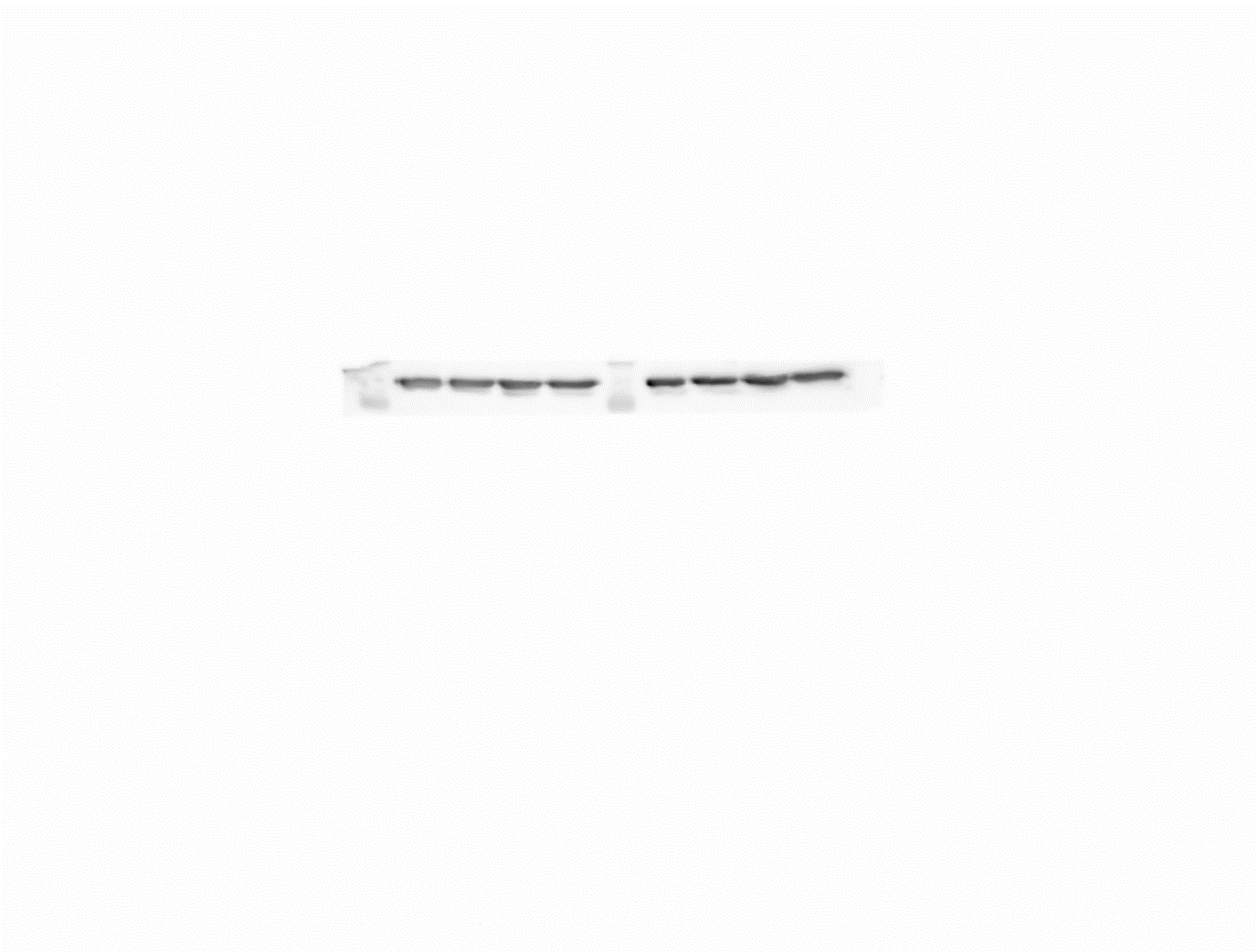


**Figure 4 (b) in the manuscript.**

**ACTIN, 786-O cell lysate.** Lane 1: MW marker; Lane 2: CTRL; lane 3: 0.5 U/ml; lane 4: 1 U/ml; lane 5: 3 U/ml, lane 6: MW marker; lane 7: CTRL; lane 8: 0.5 U/ml; lane 9: 1 U/ml, lane 10: 3 U/ml.

**
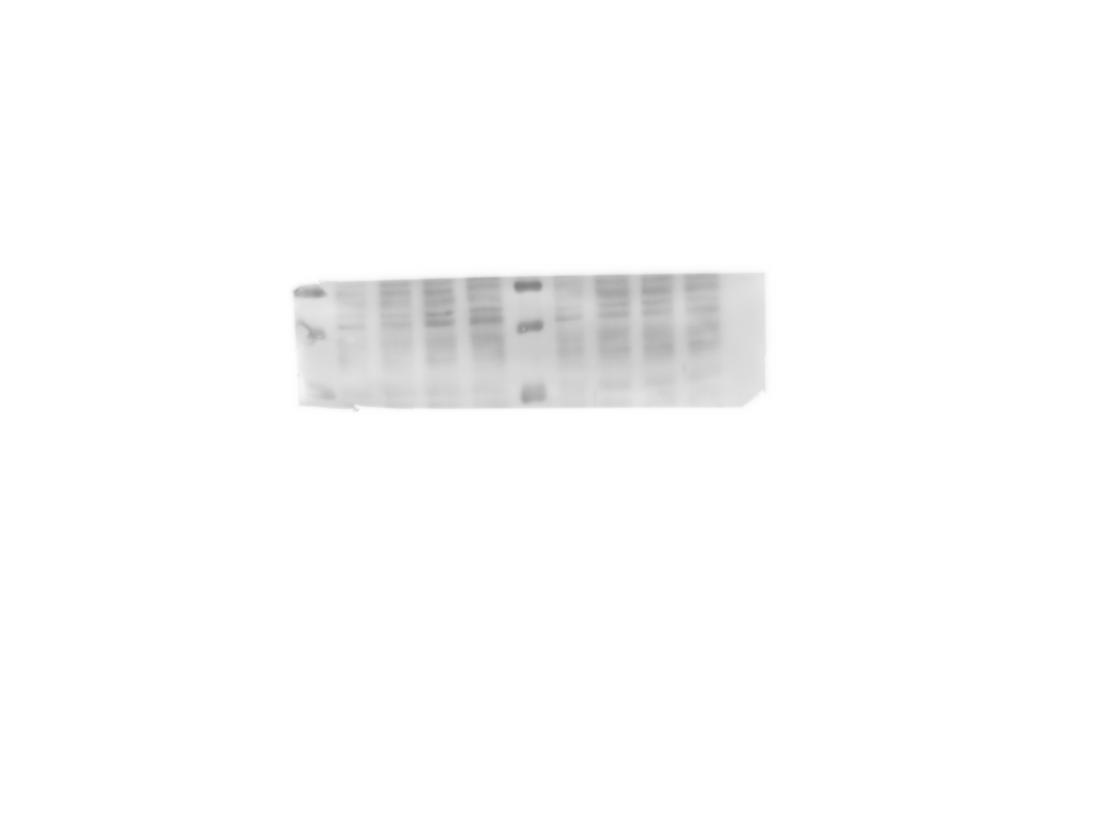
**

**Figure 4 (c) in the manuscript.**

**GS, 786-O cell lysate.** Lane 1: MW marker; Lane 2: CTRL; lane 3: 0.5 U/ml; lane 4: 1 U/ml; lane 5: 3 U/ml, lane 6: MW marker; lane 7: CTRL; lane 8: 0.5 U/ml; lane 9: 1 U/ml, lane 10: 3 U/ml.


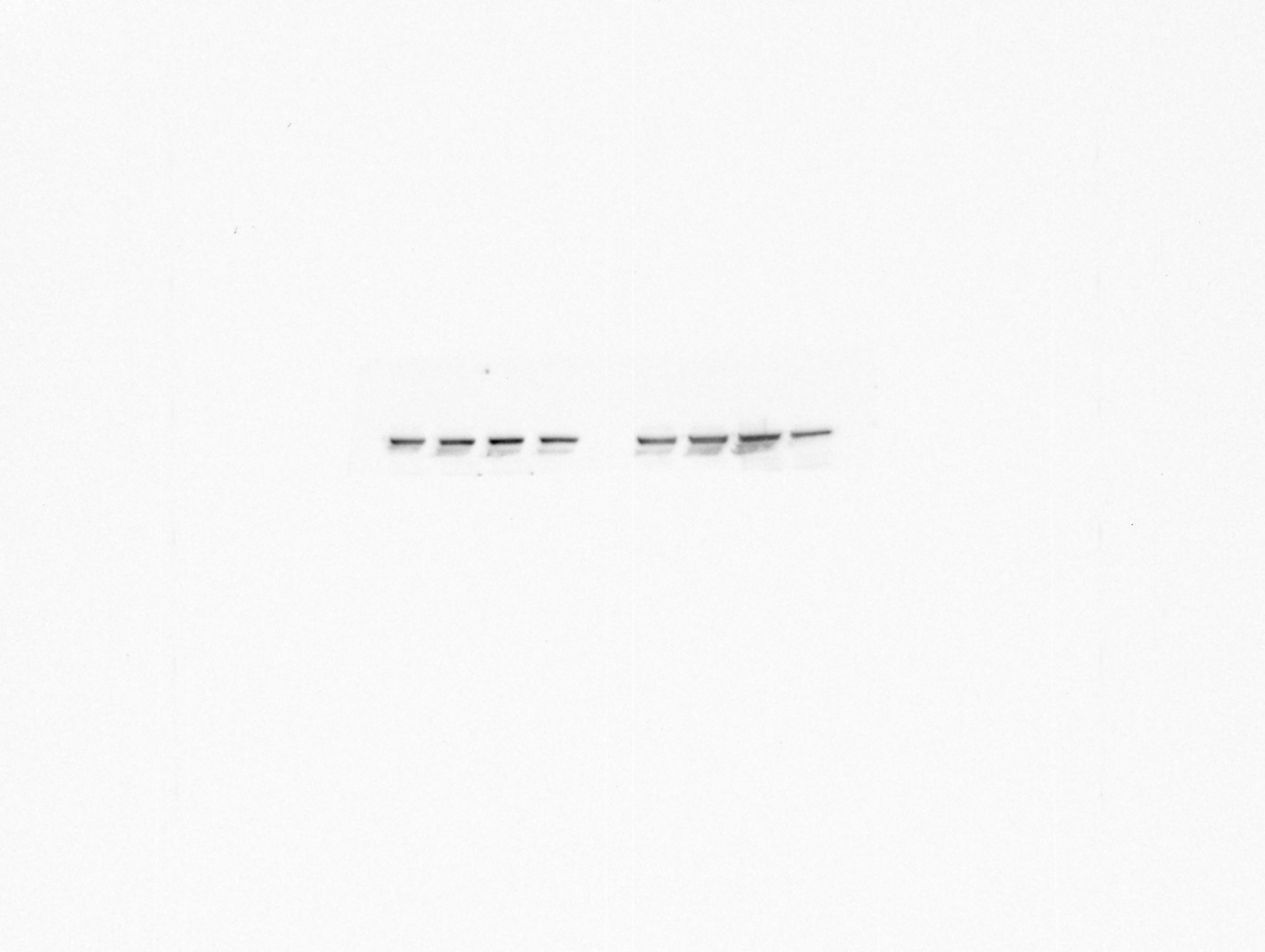


**Figure 4 (c) in the manuscript.**

**VINCULIN, 786-O cell lysate.** Lane 1: MW marker; Lane 2: CTRL; lane 3: 0.5 U/ml; lane 4: 1 U/ml; lane 5: 3 U/ml, lane 6: MW marker; lane 7: CTRL; lane 8: 0.5 U/ml; lane 9: 1 U/ml, lane 10: 3 U/ml.

**
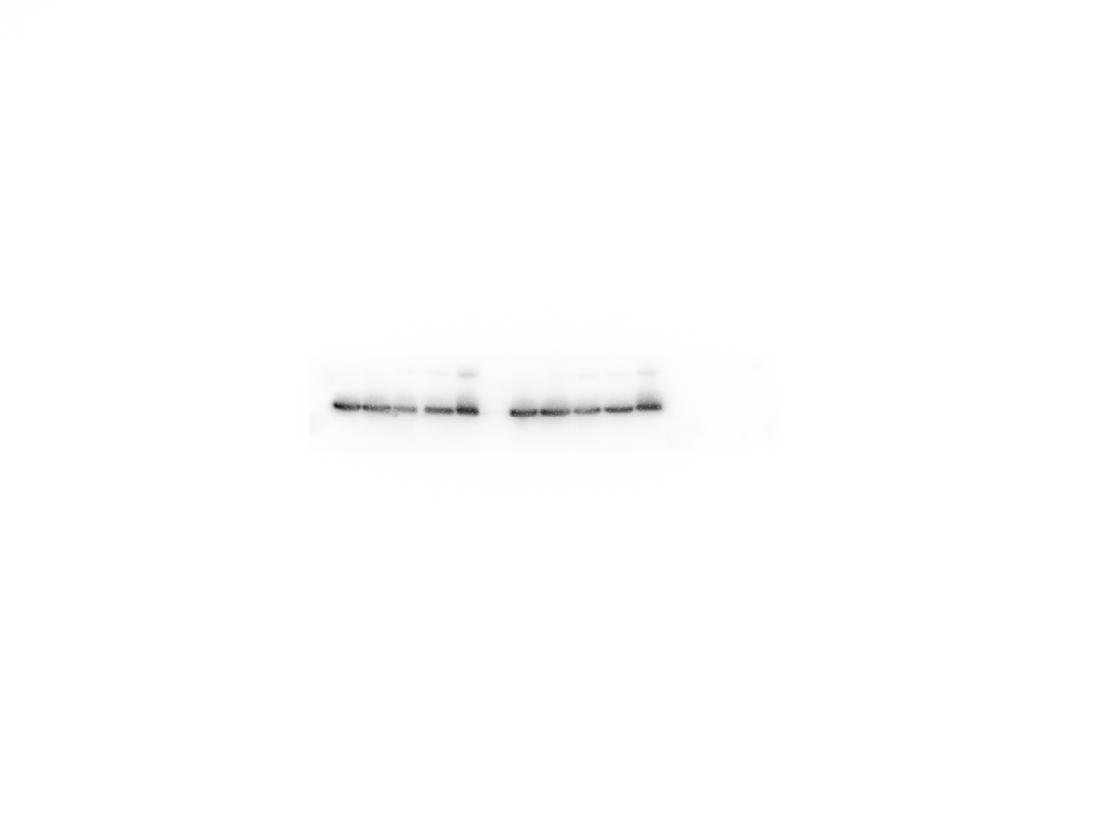
**

**Figure 4 (d) in the manuscript.**

**PCNA, A549 cell lysate.** Lane 1: MW marker; Lane 2: CTRL; lane 3: 0.05 U/ml; lane 4: 0.50 U/ml; lane 5: 1.00 U/ml, lane 6 MCF-7 (positive CTRL) lane 7: CTRL; lane 8: 0.05 U/ml; lane 9: 0.50 U/ml, lane 10: 1.00 U/ml.

**
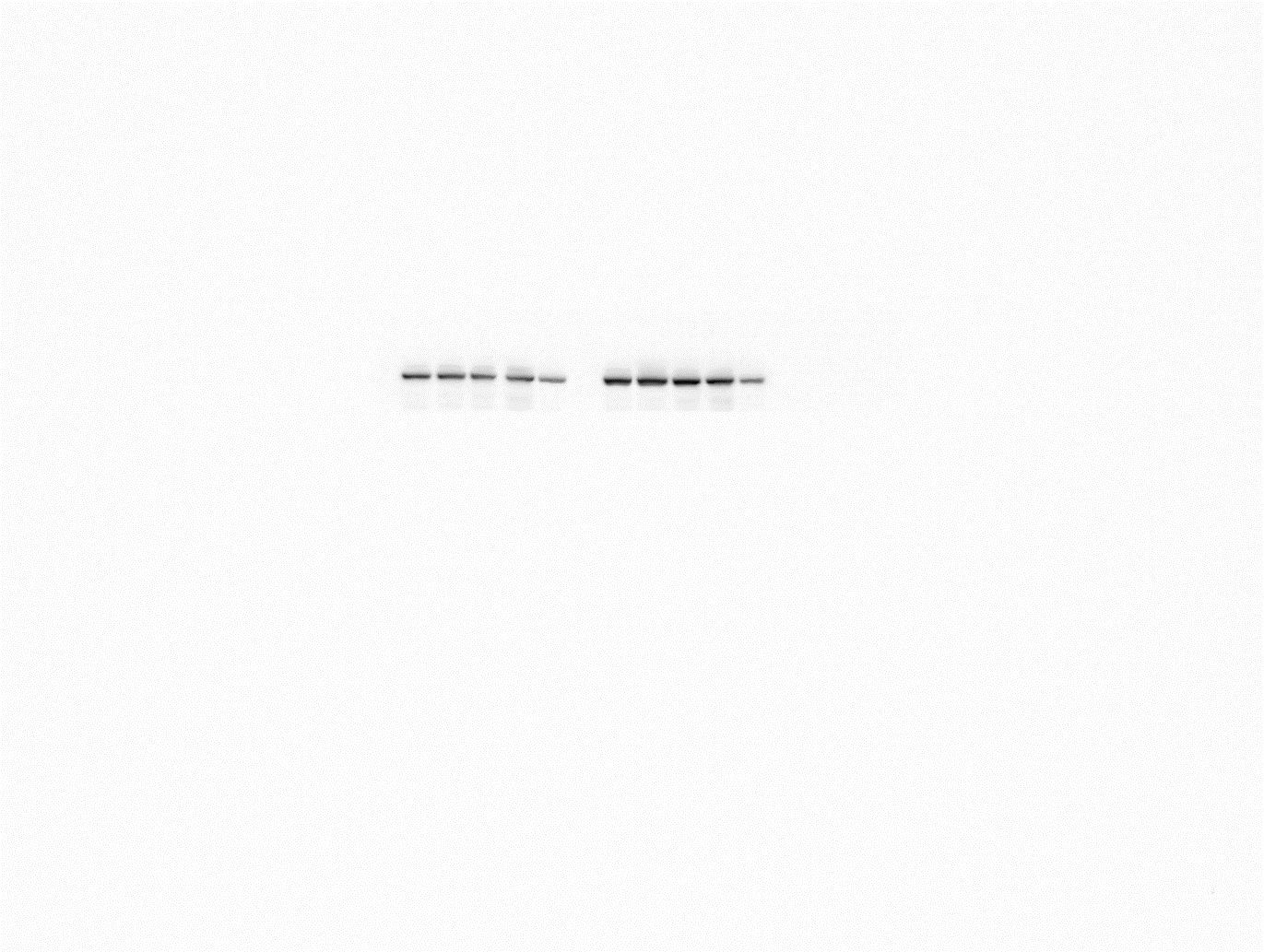
**

**Figure 4 (d) in the manuscript.**

**Vinculin, A549 cell lysate.** Lane 1: MW marker; Lane 2: CTRL; lane 3: 0.05 U/ml; lane 4: 0.50 U/ml; lane 5: 1.00 U/ml, lane 6 MCF-7 (positive CTRL) lane 7: CTRL; lane 8: 0.05 U/ml; lane 9: 0.50 U/ml, lane 10: 1.00 U/ml.

**
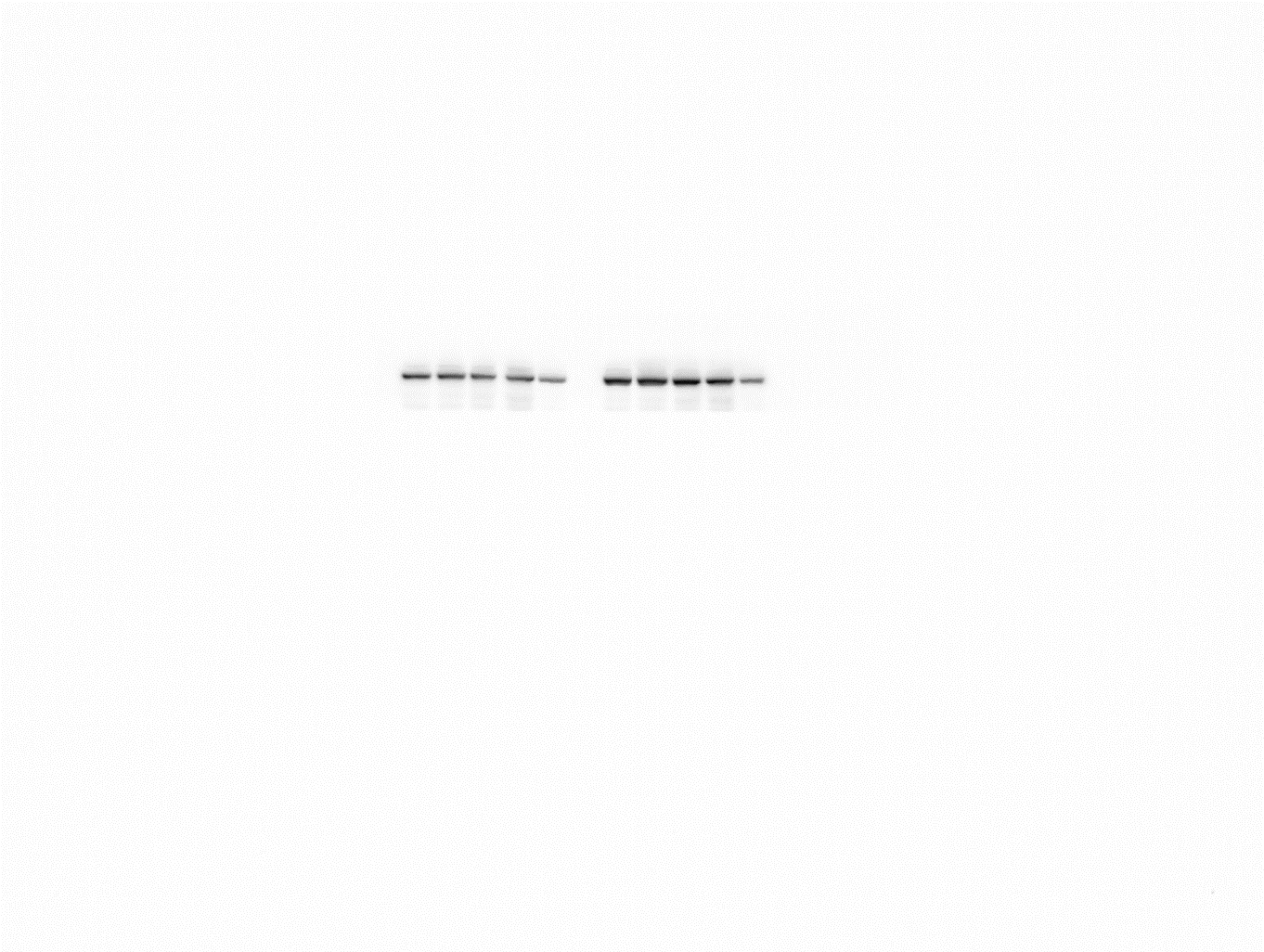
**

**Figure 4 (e) in the manuscript.**

**ASNS, A549 cell lysate.** Lane 1: MW marker; Lane 2: CTRL; lane 3: 0.05 U/ml; lane 4: 0.50 U/ml; lane 5: 1.00 U/ml, lane 6 MCF-7 (positive CTRL) lane 7: CTRL; lane 8: 0.05 U/ml; lane 9: 0.50 U/ml, lane 10: 1.00 U/ml.

**
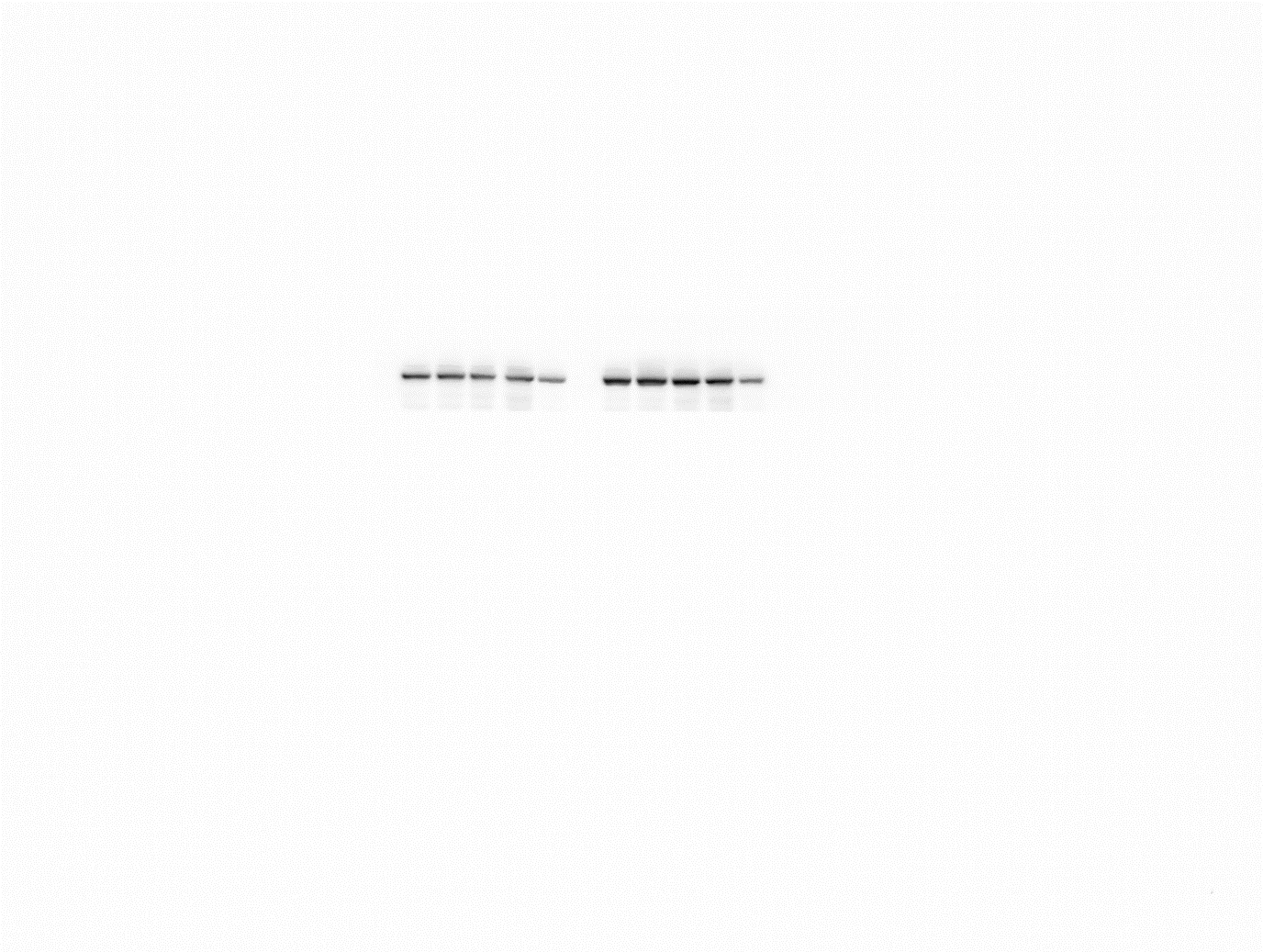
**

**Figure 4 (e) in the manuscript.**

**Vinculin, A549 cell lysate.** Lane 1: MW marker; Lane 2: CTRL; lane 3: 0.05 U/ml; lane 4: 0.50 U/ml; lane 5: 1.00 U/ml, lane 6 MCF-7 (positive CTRL) lane 7: CTRL; lane 8: 0.05 U/ml; lane 9: 0.50 U/ml, lane 10: 1.00 U/ml.

**
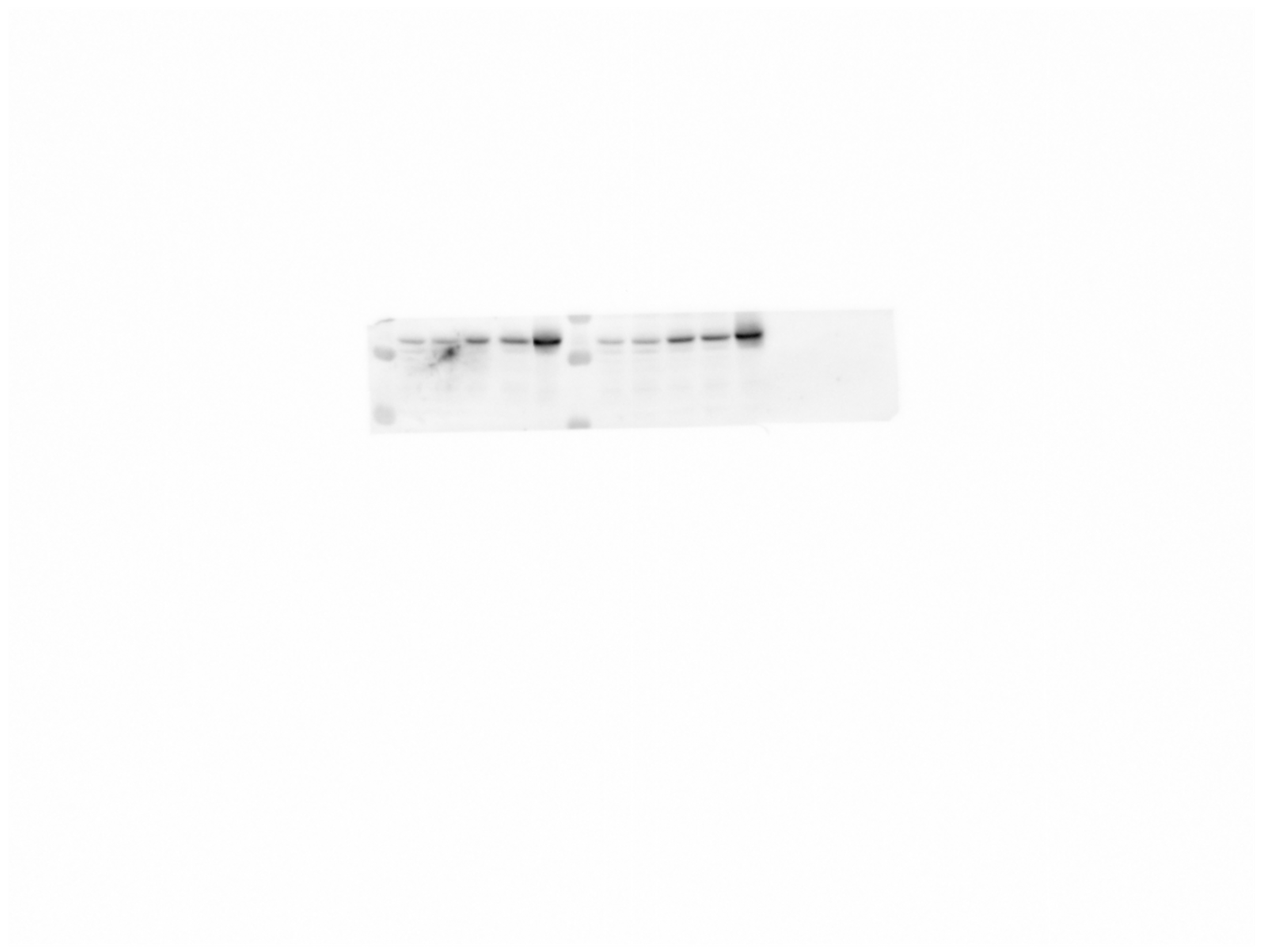
**

**Figure 4 (f) in the manuscript.**

**GS, A549 cell lysate.** Lane 1: MW marker; Lane 2: CTRL; lane 3: 0.05 U/ml; lane 4: 0.50 U/ml; lane 5: 1.00 U/ml, lane 6 MCF-7 (positive CTRL) lane 7: CTRL; lane 8: 0.05 U/ml; lane 9: 0.50 U/ml, lane 10: 1.00 U/ml.

**
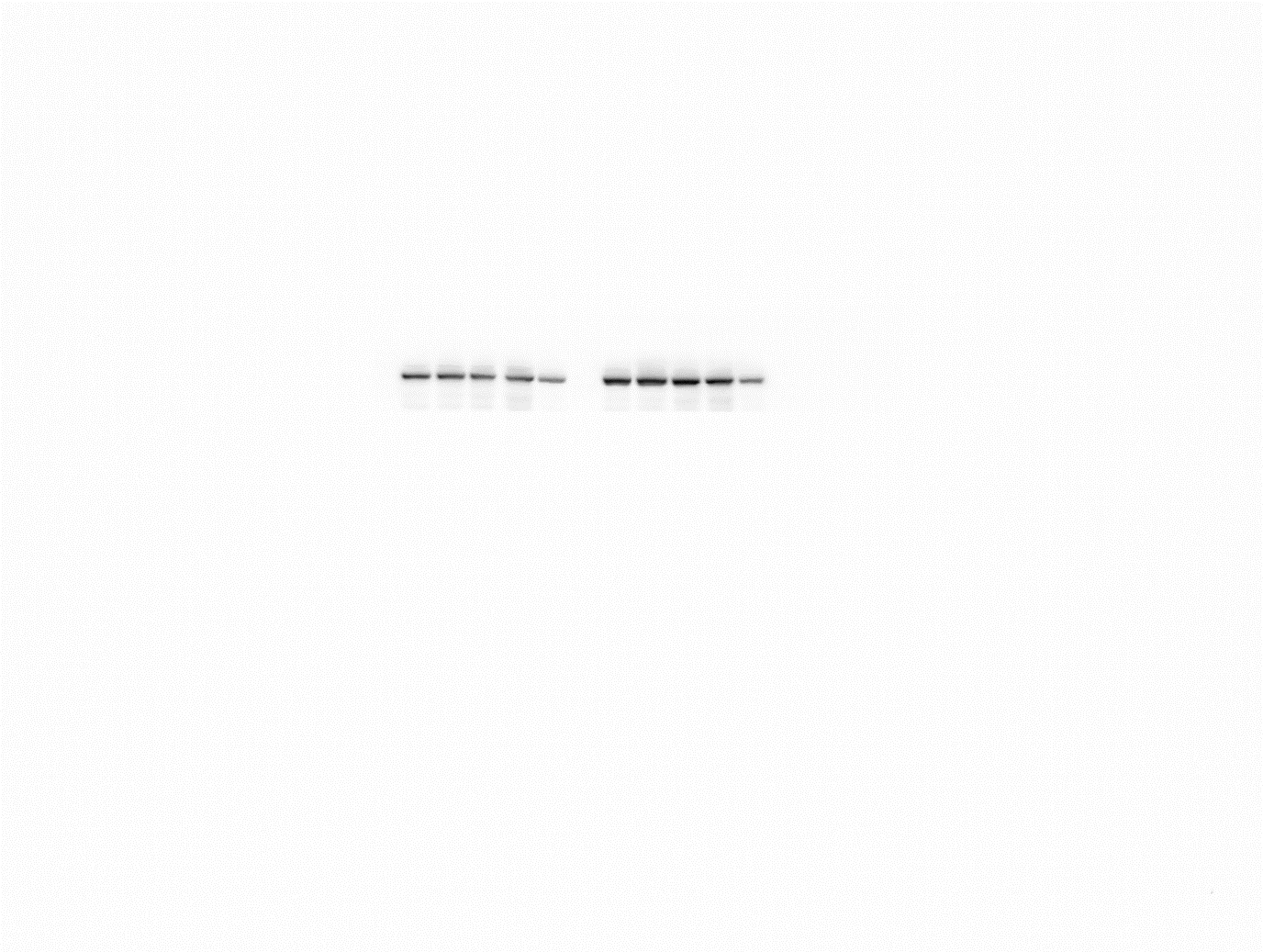
**

**Figure 4 (f) in the manuscript.**

**Vinculin, A549 cell lysate.** Lane 1: MW marker; Lane 2: CTRL; lane 3: 0.05 U/ml; lane 4: 0.50 U/ml; lane 5: 1.00 U/ml, lane 6 MCF-7 (positive CTRL) lane 7: CTRL; lane 8: 0.05 U/ml; lane 9: 0.50 U/ml, lane 10: 1.00 U/ml.

**
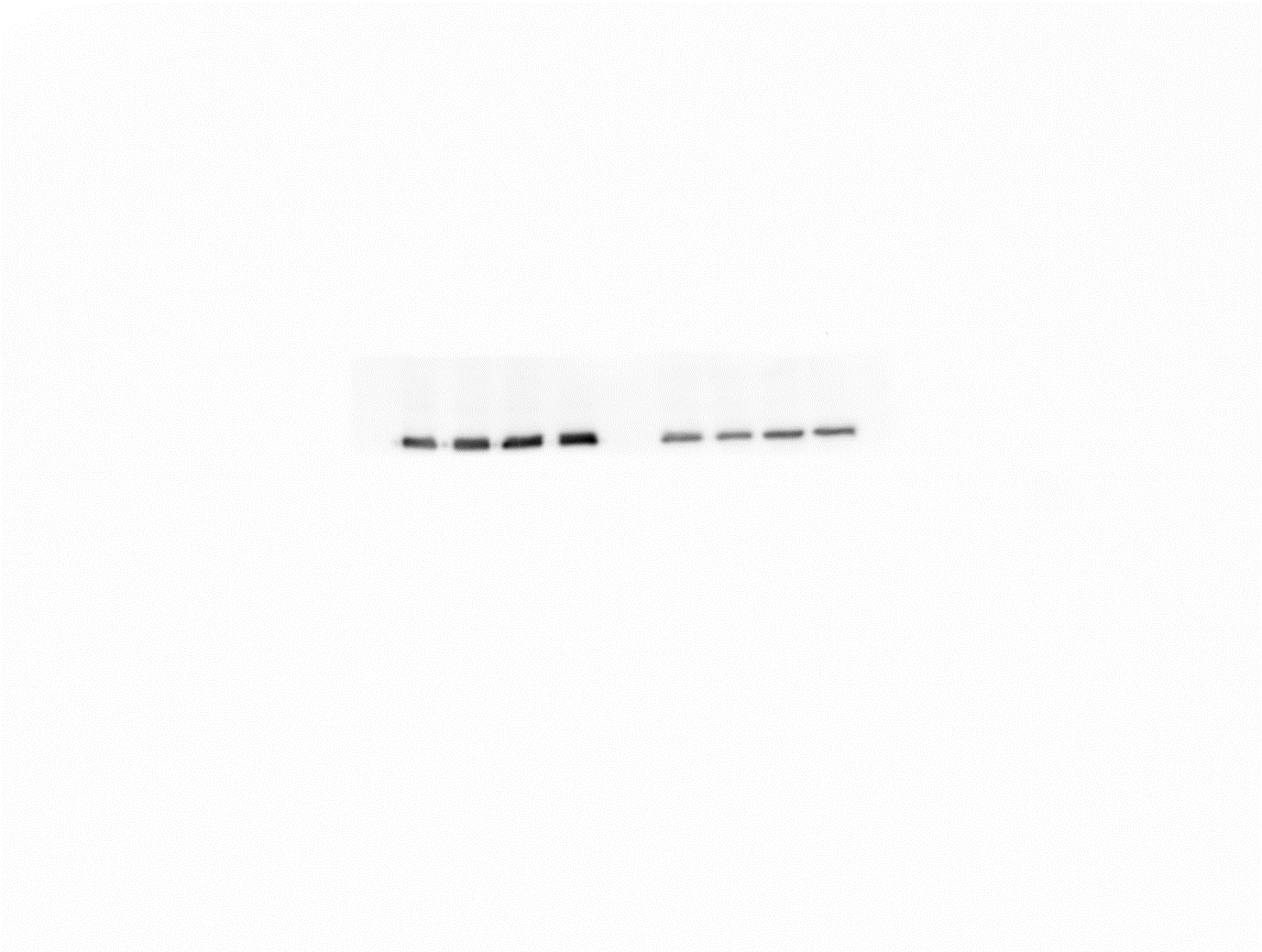
**

**Figure 4 (g) in the manuscript.**

**PCNA, MCF-7 cell lysate.** Lane 1: MW marker; Lane 2: CTRL; lane 3: 0.5 U/ml; lane 4: 1 U/ml; lane 5: 3 U/ml, lane 6: MW marker; lane 7: CTRL; lane 8: 0.5 U/ml; lane 9: 1 U/ml, lane 10: 3 U/ml.

**
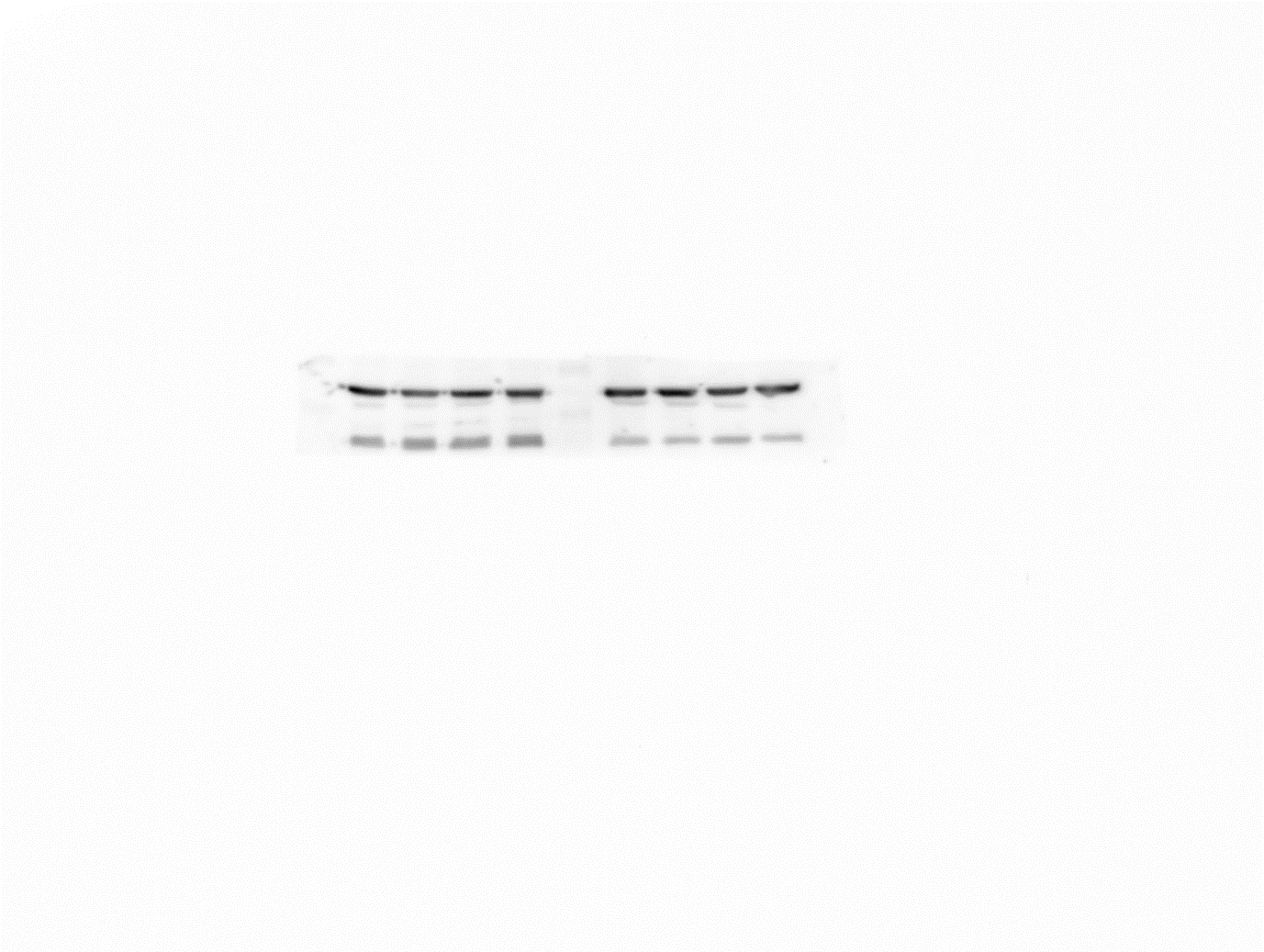
**

**Figure 4 (g) in the manuscript.**

**ACTIN, MCF-7 cell lysate.** Lane 1: MW marker; Lane 2: CTRL; lane 3: 0.5 U/ml; lane 4: 1 U/ml; lane 5: 3 U/ml, lane 6: MW marker; lane 7: CTRL; lane 8: 0.5 U/ml; lane 9: 1 U/ml, lane 10: 3 U/ml.

**
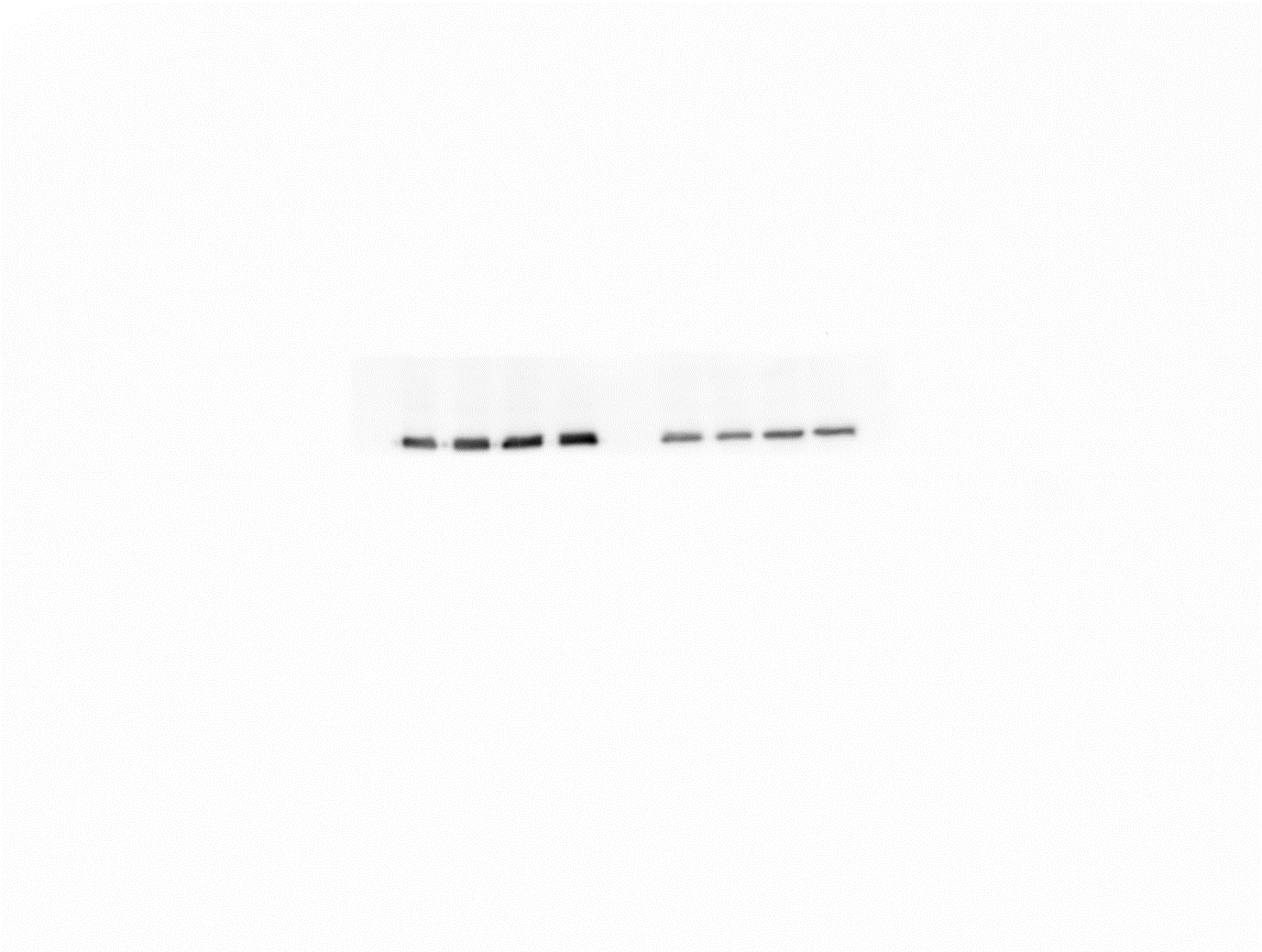
**

**Figure 4 (h) in the manuscript.**

**ASNS, MCF-7 cell lysate.** Lane 1: MW marker; Lane 2: CTRL; lane 3: 0.5 U/ml; lane 4: 1 U/ml; lane 5: 3 U/ml, lane 6: MW marker; lane 7: CTRL; lane 8: 0.5 U/ml; lane 9: 1 U/ml, lane 10: 3 U/ml.

**
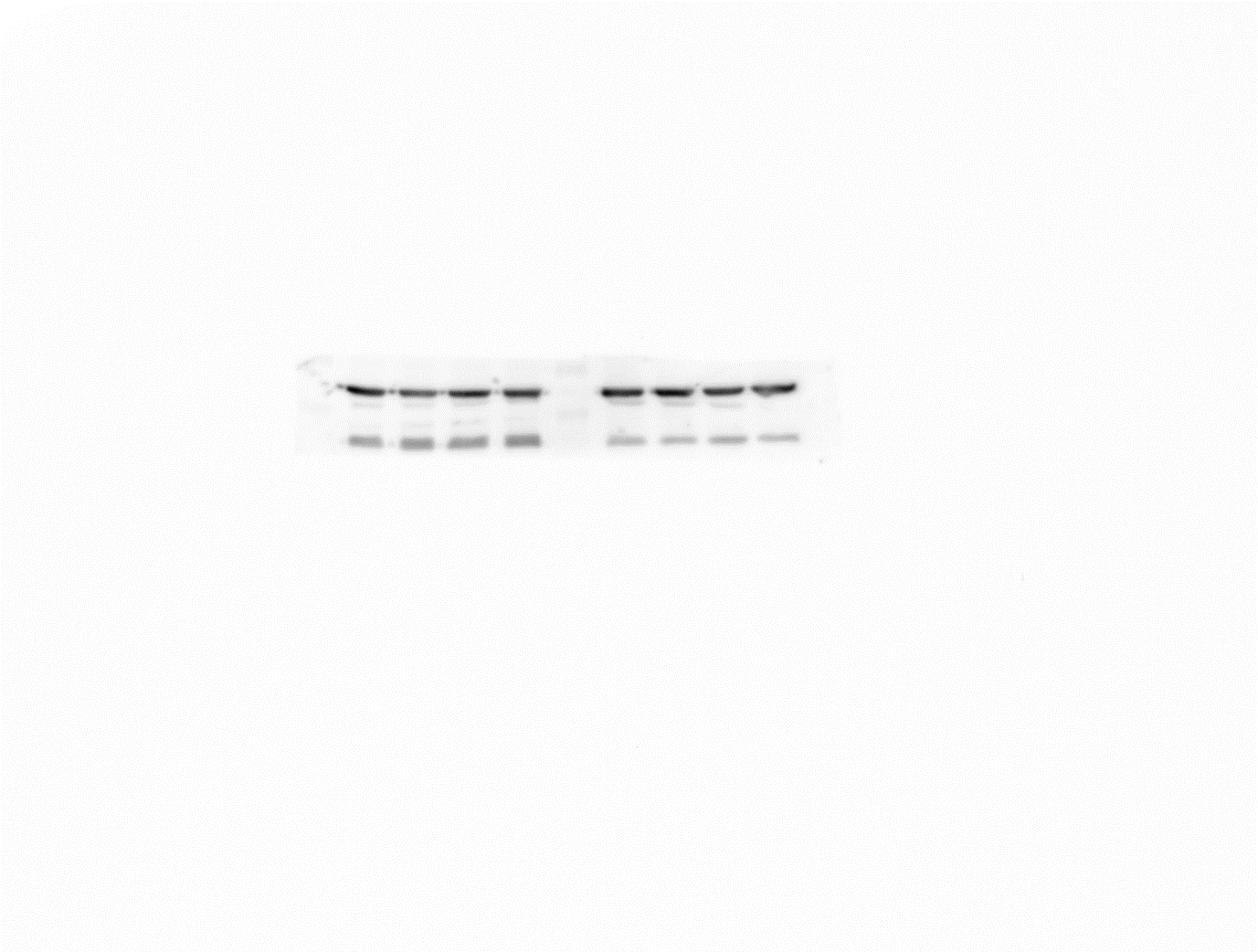
**

**Figure 4 (h) in the manuscript.**

**ACTIN, MCF-7 cell lysate.** Lane 1: MW marker; Lane 2: CTRL; lane 3: 0.5 U/ml; lane 4: 1 U/ml; lane 5: 3 U/ml, lane 6: MW marker; lane 7: CTRL; lane 8: 0.5 U/ml; lane 9: 1 U/ml, lane 10: 3 U/ml.

**
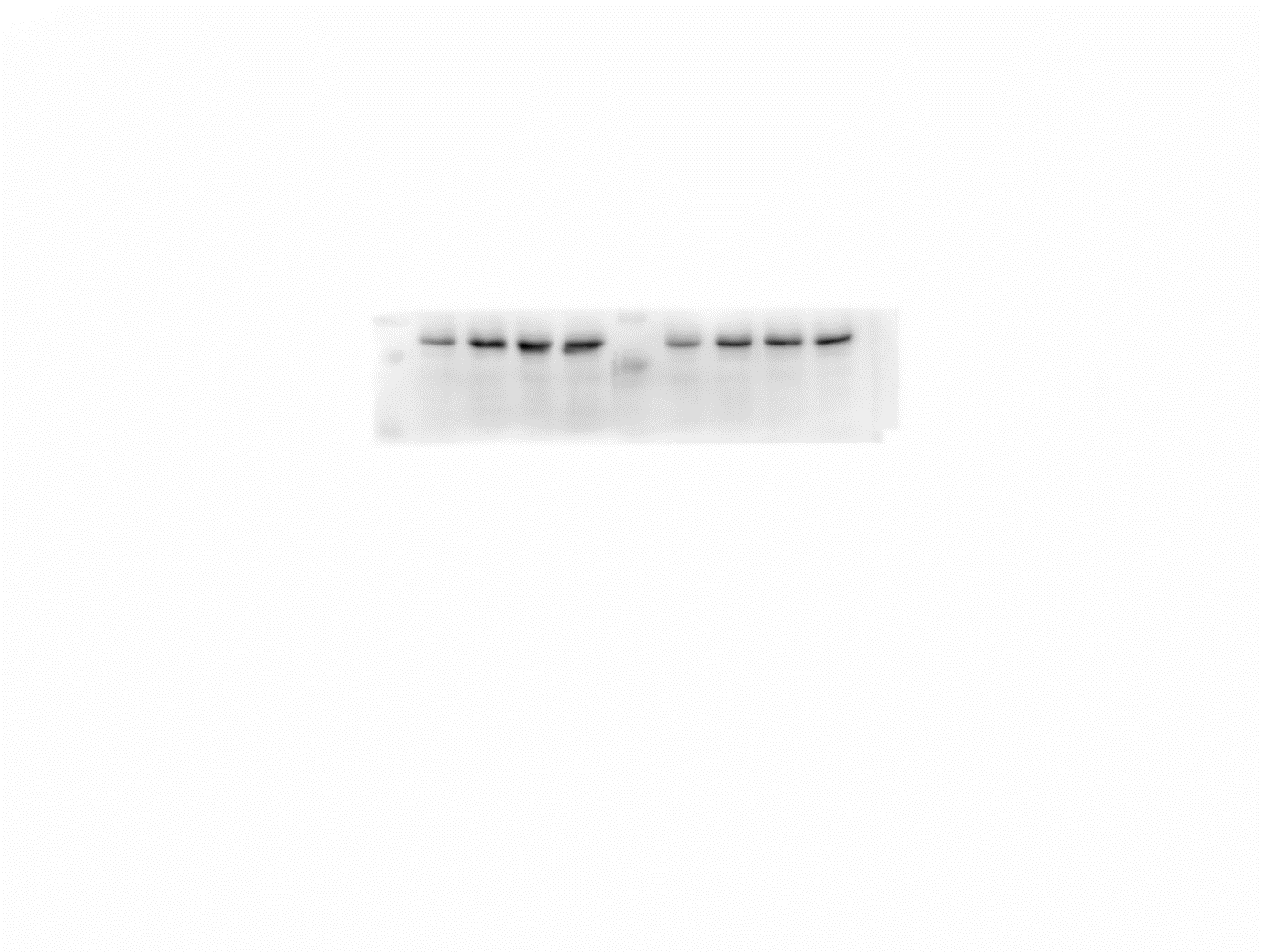
**

**Figure 4 (i) in the manuscript.**

**GS, MCF-7 cell lysate.** Lane 1: MW marker; Lane 2: CTRL; lane 3: 0.5 U/ml; lane 4: 1 U/ml; lane 5: 3 U/ml, lane 6: MW marker; lane 7: CTRL; lane 8: 0.5 U/ml; lane 9: 1 U/ml, lane 10: 3 U/ml.

**
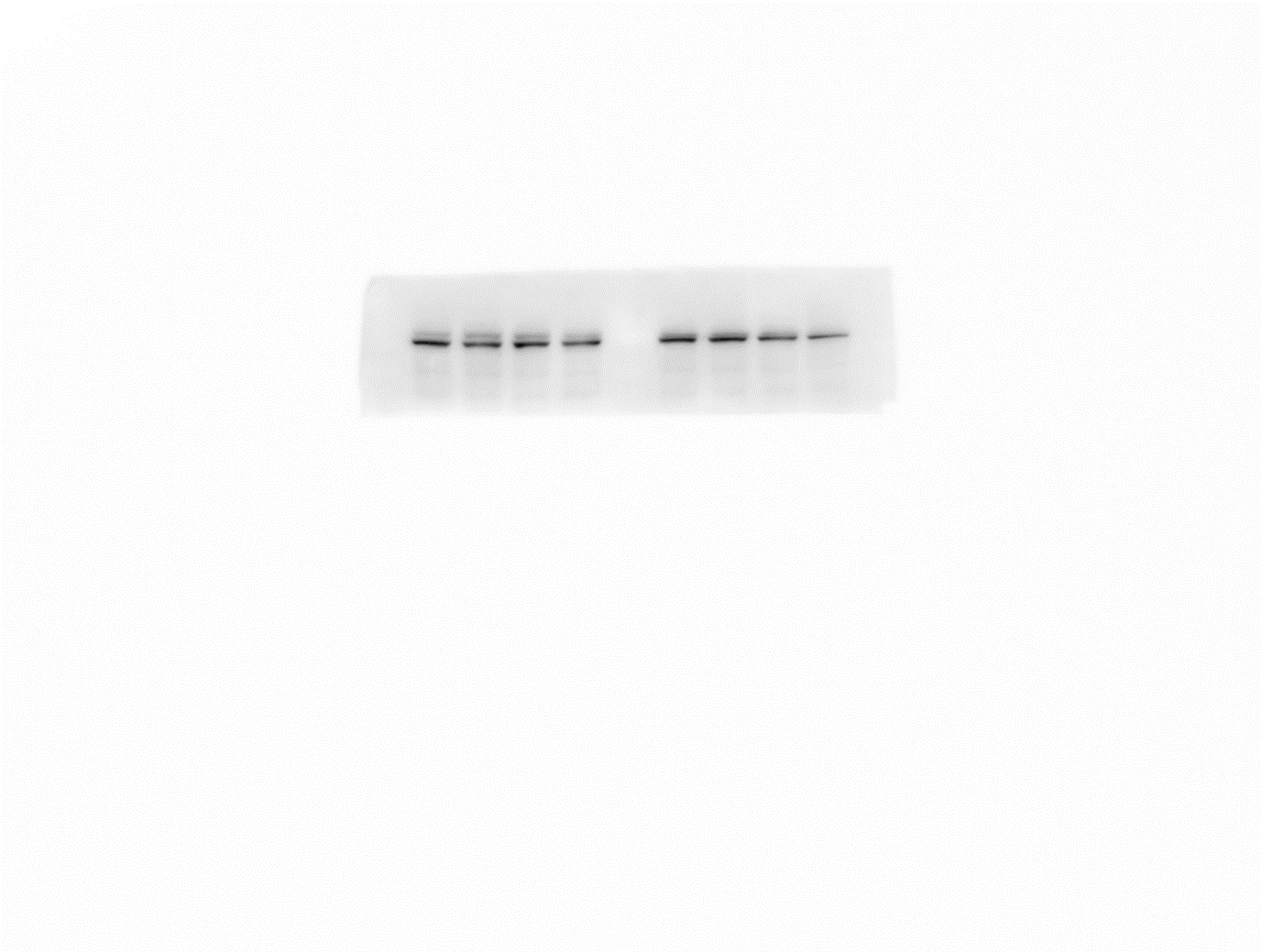
**

**Figure 4 (i) in the manuscript.**

**VINCULIN, MCF-7 cell lysate.** Lane 1: MW marker; Lane 2: CTRL; lane 3: 0.5 U/ml; lane 4: 1 U/ml; lane 5: 3 U/ml, lane 6: MW marker; lane 7: CTRL; lane 8: 0.5 U/ml; lane 9: 1 U/ml, lane 10: 3 U/ml.
